# Myeloid cells in chronic liver inflammation

**DOI:** 10.1038/s41423-025-01324-4

**Published:** 2025-07-28

**Authors:** Dimitrios Patseas, Ahmed El-Masry, Zuobin Liu, Prakash Ramachandran, Evangelos Triantafyllou

**Affiliations:** 1 Section of Hepatology and Gastroenterology, Division of Digestive Diseases, Department of Metabolism, Digestion and Reproduction, Imperial College London, London, UK; 2 Centre for Inflammation Research, Institute for Regeneration and Repair, Edinburgh BioQuarter, University of Edissnburgh, Edinburgh, UK

**Keywords:** Neutrophils, Macrophages, Dendritic cells, Liver inflammation, Liver fibrosis, Liver cancer, Chronic inflammation, Innate immunity

## Abstract

Chronic liver disease represents a significant global health burden. Regardless of etiology, its pathogenesis is driven by persistent liver inflammation, which can lead to fibrosis, cirrhosis, and an increased risk of cancer development. Myeloid cells, including neutrophils, eosinophils, monocytes, macrophages, and dendritic cells, play diverse and critical roles in hepatic immunity and the maintenance of tissue homeostasis but are also involved in liver injury, disease progression, and resolution. With the emergence of high-resolution omics technologies and in vivo fate-mapping models, our understanding of myeloid cell ontogeny and functional heterogeneity has been significantly refined. In this review, we discuss current insights into the myeloid cell landscape in nonviral chronic liver inflammatory conditions and summarize the roles of myeloid cell subsets in disease pathogenesis.

## Introduction

Liver disease is a major global healthcare burden, with estimates suggesting that over 840 million people worldwide suffer from chronic liver disease (CLD), which is associated with a mortality rate of ~2 million deaths per year [1]. Recent data indicate a rising prevalence of CLD, mainly due to the increasing incidence of metabolic dysfunction-associated steatotic liver disease (MASLD) and alcohol-associated liver disease (ALD) [2–4]. As such, there is increasing interest in identifying factors that contribute to disease development and progression.

CLD encompasses a range of etiologies, each highlighted by its unique pathogenesis but underlined by the premise that persistent liver inflammation begs fibrosis, progression to end-stage scarring termed cirrhosis, and increased risk of cancer development. MASLD, which is globally recognized as the fastest growing cause of CLD, particularly in the Western world (with a prevalence of 25–30%) [5, 6], is characterized by the development of steatosis (fat accumulation in the liver) in the presence of metabolic risk factors, such as obesity and type 2 diabetes. MASLD encompasses a spectrum of conditions, ranging from benign steatosis to a more aggressive, inflammatory form named metabolic dysfunction-associated steatohepatitis (MASH), which is associated with fibrosis and cirrhosis development [7]. ALD refers to liver disease in the context of sustained alcohol misuse. Similarly, this can be associated with benign steatosis, steatohepatitis and acutely, severe alcohol abuse and alcohol-associated hepatitis (AH) [3]. In ALD and AH, the inflammatory milieu is complex and is dictated by direct alcohol-induced hepatotoxicity, increased gut permeability leading to increased bacterial translocation, and innate immune dysregulation [8, 9].

Autoimmune hepatitis (AIH) is characterized by the presence of autoantibodies and histologic interface hepatitis, with a variable clinical presentation (asymptomatic, acute liver failure, or CLD) [10]. Liver diseases characterized by major bile duct alterations, or cholangiopathies, include primary biliary cholangitis (PBC) and primary sclerosing cholangitis (PSC), both of which are of idiopathic origin. PBC is a chronic inflammatory autoimmune disease characterized by cholestasis and progressive small bile duct cholangitis [11], whereas PSC encompasses an inflammatory cholangiopathy of largely unknown cause [12, 13], variably affecting both the small and large bile ducts depending on the subtype.

Chronic liver injury, regardless of etiology, leads to persistent activation of hepatic immune responses and subsequent inflammation. This in turn leads to aberrant activation of extracellular matrix (ECM)-producing cells such as hepatic stellate cells (HSCs) [14] and the deposition of fibrillar matrix proteins, including collagens, causing self-perpetuating liver inflammation and, as the disease progresses, cirrhosis. If left untreated or if the underlying causative factor is not removed, this may lead to the development of liver cancer, particularly hepatocellular carcinoma (HCC) or intrahepatic cholangiocarcinoma (iCCA) [15, 16]. Undoubtedly, the immune system plays significant roles in CLD development and progression. In this review, we discuss the current understanding of the functional heterogeneity and roles of neutrophils, eosinophils, monocytes, macrophages, and dendritic cells (DCs) in various nonviral chronic inflammatory liver diseases.

## The liver: a central immunological organ

**Fig. 1:**
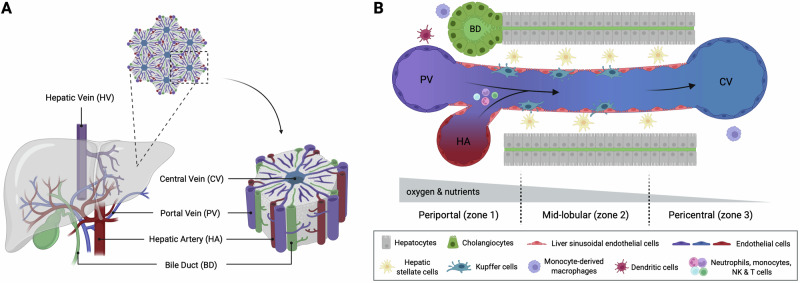
The liver is an immunological organ. **A** (Left) Macroscopic view of the liver: it receives a dual blood supply, with nutrient-rich blood from the portal vein (PV) and oxygenated blood from the hepatic artery (HA); the blood exits through the hepatic vein (HV). (Right) Schematic view of the hepatic lobules: the repeated hexagonal anatomical units of the liver. Each lobule consists of hepatocytes (main parenchymal cells) arranged around a central vein (CV) and is demarcated by a portal triad, containing a PV, an HA, and a bile duct (BD), at each corner. **B** Schematic view of liver cell subsets under homeostatic conditions. Nonparenchymal cells include immune cells, liver sinusoidal endothelial cells (LSECs), and hepatic stellate cells (HSCs). The HA and PV converge at the portal triads, and the resulting mixed blood flows through the sinusoids toward the CV, generating gradients of oxygen and nutrients that drive the functional zonation of the parenchyma into three distinct zones: periportal, midlobular, and pericentral. Blood immune cells enter and mix in the sinusoids; some cells may circulate, temporarily patrol the sinusoids and parenchyma, or become tissue-resident. Kupffer cells (KCs), the most abundant immune cell subset involved in homeostasis, reside within the sinusoids in close contact with LSECs and extend protrusions that enable interactions with HSCs (residing in the space of Disse) and hepatocytes. This position enables KCs to recognize antigens and pathogens and maintain immune tolerance. Bone marrow monocyte-derived macrophage subsets can also be found around BDs and CVs. Dendritic cells are located primarily periportally. This figure was created with BioRender (biorender.com)

The liver is the largest solid organ in the body. It is conventionally considered a nonimmunological organ that is primarily responsible for lipid metabolism, nutrient storage, and detoxification. To fulfill its physiological functions, the liver is primarily composed of hepatocytes, which are parenchymal cells that account for ~60–70% of the total cell count and 80% of the liver mass [17, 18]. The remaining cells are nonparenchymal cells, including endothelial cells, HSCs, Kupffer cells (KCs), and other immune cell populations [17, 18]. Anatomically, the liver is organized into repeating hexagonal lobules, each outlined by six “portal triads”, which consist of a bile duct, portal venule and hepatic arteriole (Fig. 1A). The liver has a dual blood supply: ~80% of the blood originates from the gut and flows via the portal vein, where it carries nutrients and gut-derived molecules, whereas the remaining 20% is oxygenated arterial blood delivered through the hepatic artery. These blood supplies mix within specialized capillaries known as liver sinusoids, which are lined with liver sinusoidal endothelial cells (LSECs) and drain into the central vein [19].

The unique structure of liver sinusoids, characterized by fenestrated endothelial cells lacking a basement membrane, facilitates low-pressure blood flow, enabling direct interactions between immune cells and hepatocytes, as well as the exchange of substances. Variations in the microenvironment, including Wnt signaling, oxygen levels, and nutrient gradients, give rise to functional liver zonation [20, 21], with the hepatic lobule divided into three zones: periportal (zone 1), mid-lobular (zone 2) and pericentral (zone 3) [17, 21, 22]. Functional zonation has been described in hepatocytes [23–25], LSECs [26], HSCs [27], and immune cells such as KCs [28–30].

The liver plays a critical role in immune surveillance and tissue homeostasis because of its strategic location at the confluence of the dual blood supply (Fig. 1A) [31, 32]. As the gut serves as a major entry point for pathogens, the liver must recognize, neutralize, and clear gut-derived pathogens before the blood re-enters the systemic circulation [32, 33]. This process requires a delicate balance between immune activation—targeting harmful pathogens—and tolerance—toward harmless molecules such as dietary antigens—to prevent inappropriate inflammation [33, 34]. This finely tuned homeostatic equilibrium is tightly regulated and essential for maintaining proper liver function.

Under homeostatic conditions, the liver is predominantly populated by innate immune cells, including macrophages, DCs, and natural killer (NK) cells [32, 34] (Fig. 1B). Among these, KCs, which are liver-resident macrophages, serve as key effector cells responsible for detecting microbial molecules, clearing pathogens, and producing cytokines and chemokines to mediate inflammatory responses [35, 36]. Numerous studies have demonstrated that KCs play a vital role in controlling and resolving infections caused by bacteria such as *Staphylococcus aureus* and *Escherichia coli* (*E. coli*) [28, 37–48].

The ability of the liver to recognize and neutralize danger signals relies on the broad expression of pattern recognition receptors (PRRs), including toll-like receptors (TLRs) and NOD-like receptors [49, 50]. These receptors are expressed by KCs, hepatocytes, and professional antigen-presenting cells (APCs), allowing them to detect a variety of pathogen-associated molecular patterns (PAMPs) from microorganisms and damage-associated molecular patterns (DAMPs) from host tissue damage. Overall, the liver plays a key role as an immunological organ, particularly at the interface between the gut and systemic circulation, and is critical for host defense.

## Myeloid cells in chronic liver inflammation

Myeloid cells constitute a significant proportion of leukocytes in the circulation and peripheral tissues, including the skin, lungs, gut, and liver [51]. They include granulocytes (neutrophils, eosinophils, and basophils), monocytes, macrophages, and DCs. These cells represent major cellular compartments of the innate immune system and play key roles in antimicrobial defense and inflammation, as well as in tissue development, homeostasis, and repair. With the advent of high-resolution single-cell omics technologies and in vivo fate-mapping models, the fields of myeloid cell ontogeny, heterogeneity, maturation, and specialization in homeostasis and inflammatory diseases [51–56], including cancer [57, 58], have been extensively revised. Recent studies suggest that, beyond their origin, a multitude of diverse microenvironmental cues can influence myeloid fate and function, with increasing evidence indicating that granulocytic subsets, such as neutrophils and eosinophils, are more heterogeneous than previously appreciated. This article provides a comprehensive overview of the phenotypical and functional properties of neutrophils [59], eosinophils [60], monocytes/macrophages [61–67], and DCs [68–72] in the context of liver health and disease, highlighting key discoveries.

### Neutrophils

#### Origin and functions of neutrophils

Neutrophils, the predominant type of circulating leukocyte, are endowed with antipathogenic and inflammatory effector functions and are therefore critical for maintaining host immunity and tissue homeostasis [73, 74]. The multifaceted activities of these cells underscore their remarkable phenotypic and functional diversity [75] under both steady-state and stress conditions. As explored below, neutrophils were previously thought to be transcriptionally silent, terminally differentiated cells; however, this view has recently been challenged, and neutrophil plasticity is now widely recognized.

Neutrophils develop through granulopoiesis, an intricately regulated process by which immune cells in the bone marrow undergo progressive modifications to their transcriptomic, proteomic, structural, and consequently functional properties before being released into the circulation [52]. Under homeostatic and emergency conditions, granulopoiesis directs neutrophil differentiation and development from hematopoietic stem cells to common myeloid progenitors (CMPs), then to granulocyte‒macrophage progenitors (GMPs), and subsequently to lineage-specific maturation. Neutrophil commitment can be divided into two stages: (1) determination: a stage of differentiation to preneutrophils, after which only effector functions can be acquired, and (2) specification: whereby the neutrophil lineage is committed to, in the form of pro-neutrophils [52]. This process of specification, alongside the concurrent development of effector functions, is tightly guided by granulocytic transcription factors, primarily growth factor independence-1 (GFI-1) and CCAAT-enhancer binding protein-ε (C/EBPε) [52, 76]. Functional maturation is preestablished through pro-neutrophil chromatin remodeling, with specific effector roles peaking at different stages of subsequent maturation [74].

In the event of emergency granulopoiesis triggered by physiological stress, de novo neutrophil generation occurs [77]. Noncommitted precursor and immature committed low-density neutrophils (LDNs) expand and are prematurely released into the circulation, where they coexist with terminally differentiated subsets. In this context, these early neutrophil lineage subsets exhibit altered effector function capabilities compared with their mature counterparts [52, 74]. Heterogeneity under steady-state and stress conditions has thus been described, with phenotypic and functional alterations ultimately determined by physiological and pathologic contexts [74]. These contexts are highly dynamic and are shaped by combined influences such as homeostatic fluctuations (e.g., granulopoiesis state origin) [74], local bone marrow niche composition [78, 79], circadian rhythm [80, 81], stage of maturation postspecification, and the local microenvironment (circulating [82] or tissue-resident [83]). Further research is now needed to explore heterogeneity within individual circulating and tissue-resident neutrophil subsets.

Neutrophils are among the first innate immune cells to mobilize to sites of tissue injury, orchestrating the host immune response during inflammation while also coordinating critical antipathogen responses. They perform several antipathogen effector functions that, even in a state of health, vary depending on neutrophil density (determined by nuclear morphology and granule content), maturation stage, or activation state [74] as described below. Chemotaxis and adhesion are closely interlinked processes that enable neutrophils to respond effectively to sites of injury or infection. Chemotaxis involves their migration toward chemoattractants, such as chemokines, DAMPs and PAMPs [59] while adhesion is facilitated by proinflammatory agents [e.g., interleukin-1 (IL-1), IL-17, and tumor necrosis factor alpha (TNF-α)], which increase vascular permeability and stimulate the expression of adhesion molecules on the endothelium (selectins and integrins) and constitutively expressed receptors on neutrophils (P-selectin ligand 1 and L-selectin) [9, 84]. Together, these processes ensure that neutrophils can not only locate sites of inflammation but also firmly adhere to the endothelium.

Neutrophils capture and kill pathogens through several mechanisms. They interact directly with pathogens or via receptors such as Fc receptors (FccRIIIB/CD16 and FccRIIA/CD32), integrins (CD11b/CD18), and complement receptors (CR1/CD35 and CR3) to facilitate phagocytosis [8, 9]. Once internalized, pathogens are destroyed within phagolysosomes by intracellular granules and reactive oxygen species (ROS) [9, 52, 59]. Neutrophils generate ROS primarily through nicotinamide adenine dinucleotide phosphate (NADPH) oxidase, which produces superoxide anions and hydrogen peroxide, and secondarily through free hydroxyl radicals formed by myeloperoxidase (MPO) [85, 86].

Pathogen interactions and inflammatory signals can also induce neutrophil degranulation, leading to the release of proteases, MPO, and defensins that contribute to pathogen destruction. In addition, neutrophils produce various chemokines [C-X-C motif chemokine ligand 1 (CXCL1), CXCL2, CXCL8 and CXCL12] and cytokines, including proinflammatory (IL-1α, IL-1β, IL-6, TNF-α), anti-inflammatory [IL-10, transforming growth factor beta (TGF-β)], and immunoregulatory (IL-22, IL-23) mediators [9, 52, 59]. Uncontrolled release of proinflammatory cytokines by aberrantly or persistently activated neutrophils can exacerbate local and systemic inflammation, promoting a “cytokine storm”. However, whether the degree to which neutrophil cytokine release coordinates systemic responses in a manner similar to that of other immune cells (e.g., lymphocytes) remains unclear.

Neutrophil extracellular trap (NET) formation, or NETosis, is another key function of these cells. NETs consist of extruded decondensed DNA, citrullinated histones, and a mixture of proteins of nuclear, cytosolic, and granular origin, including neutrophil elastase (NE) and MPO [9, 52, 59]. This process is ultimately driven by ROS production, wherein MPO activation leads to granule release into the cytoplasm and subsequently into the nucleus, promoting chromatin decondensation, whereas PAD4 activation induces histone citrullination [52, 87]. The decondensed DNA then mixes with the cytoplasmic granular content and is ultimately released into the surrounding environment following disintegration of the neutrophil plasma membrane. NETs are produced to capture, immobilize, and kill pathogens.

In homeostasis, the effector functions of neutrophils are well-regulated and harmoniously coordinated, leading to the resolution of inflammation, preservation of tissue integrity through injury healing, and efficient eradication of invading pathogens. However, in states of aberrant activation or sustained inflammation, neutrophil dysfunction can prevail, resulting in tissue damage and disease progression, depending on the underlying pathology.

#### Neutrophils in chronic liver inflammation

##### MASLD

In steatotic liver disease, lipid accumulation within hepatocytes induces metabolic stress. This triggers the activation of resident immune cells and the subsequent recruitment of circulating immune cells in response to the release of proinflammatory cytokines and chemokines. Hepatic neutrophil infiltration is a hallmark of MASH development [88]. This is not only evident in human liver biopsies [89, 90] but also observed in the early stages of high-fat diet (HFD) feeding in mice [91, 92], in which neutrophil depletion via Ly6G-neutralizing antibodies abrogates metabolic syndrome development (weight gain and increased blood glucose), initial liver inflammation and injury [91, 93].

Neutrophil-derived factors such as NE and human neutrophil peptide-1 (HNP-1) can activate KCs and recruited macrophages while also inducing the proliferation of HSCs [94, 95]. Together, these effects drive inflammation and fibrogenesis. In HFD-fed mouse models, CXCL1 [96], CXCR2 (induced via lipocalin-2) [97], and IL-8-mediated neutrophil infiltration have been shown to contribute to MASH pathogenesis through various mechanisms, including granule enzyme (e.g., MPO) and ROS release and increased activation of stress kinases [90, 98]. Notably, elevated in the serum of patients with MASH [99], MPO can increase oxidative stress, contributing to injury through hepatocyte death while activating HSCs [90]. Furthermore, NE disrupts lipid homeostasis through hepatic ceramide synthesis, resulting in insulin resistance [100]. Compared with NE knockout mice, wild-type (WT) mice fed a Western diet (WD) for 24 weeks presented significantly greater weight gain, biochemical derangements (high serum lipid/triglyceride levels and transaminitis), and histological fibrosis progression [100].

Aberrant neutrophil activation further leads to NETosis, with elevated serum NET levels observed in MASH patients [8]. In HFD-fed mouse models (with/without additional alcohol insult), NET formation results in increased inflammation and fibrosis [92, 101]. Treatment with deoxyribonuclease (DNase) attenuated monocyte-derived macrophage infiltration [101] and the resulting tissue injury, although it did not halt disease progression [102]. Overall, neutrophils appear to contribute to the early pathogenesis of MASH, wherein hepatocyte injury is potentiated by inflammatory mediators, ROS, and NETs. Although a potential restorative role for neutrophils, through the regulation of the macrophage phenotype, has been described in MASH/fibrosis (as further discussed below), their precise functions across the MASLD spectrum require further investigation.

##### ALD

In ALD, neutrophil recruitment depends on multiple variables, including neutrophil priming, circulating chemokines, and the activation of LSECs [103]. Neutrophil priming and activation may be prerequisites for their recruitment into the liver parenchyma, with enhanced activation of circulating neutrophils characterized by reduced L-selectin (CD62L) expression observed in AH patients [104]. Several chemokines released from hepatocytes, HSCs, KCs, and LSECs, including CXCL1, CXCL2, and CXCL8, promote neutrophil recruitment [105, 106]. Once recruited, neutrophils adhere to the endothelium and transmigrate into the hepatic microenvironment.

In AH, gastrointestinal dysbiosis and increased gut permeability lead to a greater bacterial load in the enterohepatic circulation. Recognition of PAMPs activates neutrophils and their arsenal of antimicrobial functions. Evidence suggests that neutrophils in AH are hyperresponsive, producing increased levels of inflammatory cytokines and chemokines [107] and demonstrate increased baseline oxidative burst capacity [108]. Compared with healthy controls, NET formation has been described as a driver of chronic inflammation in AH, with high serum NE and citrullinated histone H3 (H3Cit) levels and increased spontaneous NET formation capability [109]. Interestingly, these neutrophils release fewer NETs upon phorbol myristate acetate (PMA) stimulation [109], suggesting that they are primed but exhibit a defective antimicrobial response to subsequent challenge [103]. NETs further induce monocyte differentiation into CD14^+^CD16^+^ intermediate inflammatory phenotypes and reduce macrophage efferocytosis, thereby perpetuating chronic inflammation and tissue damage [109].

Recent single-cell RNA sequencing (scRNA-seq) of hepatic neutrophils in AH has revealed an expanded IL-8^+^ subset within an already elevated total neutrophil population compared with ALD patients and healthy controls [110]. Pathway analysis revealed that the top three differentially expressed hallmark pathways in this subset were related to TNF-α, interferon gamma (IFN-γ), and inflammatory responses [110]. Given that IL-8 is key for neutrophil activation, the accumulation of IL-8^+^ neutrophils in AH may contribute to a self-sustaining proinflammatory environment, thus representing a potential therapeutic target for disease management.

Overall, in the context of AH, neutrophils exhibit a proinflammatory, typically low-density phenotype that is aberrantly activated in response to dysbiosis. This activation drives the recruitment and differentiation of various innate immune cell subtypes. When unregulated, this process perpetuates inflammation, contributing to increased morbidity and mortality.

##### AIH, PBC, and PSC

Dysregulation of immune responses to self-antigens is a hallmark of autoimmune disorders [52]. Neutrophils have been implicated in a variety of autoimmune conditions, including systemic lupus erythematosus, rheumatoid arthritis, multiple sclerosis, and inflammatory bowel disease [111]. In general, tissue-infiltrating neutrophils are involved in autoantigen presentation, the release of proinflammatory effector molecules/cytokines, and altered ROS production [111]. These multifaceted functions have also been demonstrated in immune-mediated CLD.

In AIH, the presence of an expanded population of circulating LDNs has been described [112]. The presence of NETosis, alongside dominant autoantibodies [e.g., antinuclear and anti-alpha smooth  muscle actin (α-SMA) antibodies], suggests that NETosis contributes to abnormal autoimmune responses to self-antigens, thus driving disease progression and flares [112–115]. This hypothesis is further supported by the increased presence of MPO^+^ LDNs in AIH patients compared with healthy individuals [112, 115]. As such, the presence of LDNs may serve as a biomarker indicative of disease severity. However, the extent to which NETs contribute to AIH pathogenesis and whether circulating or liver-infiltrating neutrophils are more significantly implicated remain unclear.

In biliary injury, particularly in PBC, proinflammatory cytokines, including IL-6, IL-8, and TNF-α, are released [116], promoting the recruitment and activation of innate and adaptive immune cells, as well as mesenchymal cells, to initiate biliary repair [116, 117]. IL-8 has been observed in small bile ducts in PBC patients, particularly in patients with cirrhosis [117, 118]. IL-8 positivity in early disease is relatively infrequent [117], suggesting that its persistence contributes to disease progression but may not represent the initial driver. Clinically, the neutrophil‒lymphocyte ratio (NLR) is positively associated with nonresponse to ursodeoxycholic acid (UDCA) treatment [119].

In PSC, a mixed biliary tract inflammatory cell infiltrate, including lymphocytes, plasma cells, neutrophils, NK cells, and macrophages, has been described [116, 120]. Histologically, periductular inflammation in acinar Zone 1, a process associated with marked neutrophil infiltration, is observed [116, 121]. Neutrophil infiltration has been described in the context of biliary-resident T helper 1 and T helper 17 (Th1/Th17) cell activation; these cells display a CD103^+^ CD69 effector memory phenotype with a CXCL8-associated transcriptional profile [118] and are capable of producing IL-17 and IL-22 [122]. Additionally, the chemoattraction of neutrophils is promoted by C–C motif chemokine ligand 24 (CCL24), which is released from damaged cholangiocytes [123]. Although an increased proportion of MPO^+^ neutrophils are histologically detected, the absence of MPO in mouse models [Mdr2-deficient (Mdr2^−/−^)] did not confer protection from cholangiocyte injury [124]. The precise mechanisms by which neutrophils contribute to the PSC have yet to be fully elucidated.

Overall, neutrophils have not been widely explored in patients with liver autoimmune conditions, which may be challenging because of the significant histological overlap among AIH, PBC, and PSC. Further work is needed to comprehensively evaluate phenotypic variations in circulating and liver-infiltrating neutrophils to better understand their role in disease pathogenesis.

##### Fibrosis

**Fig. 2:**
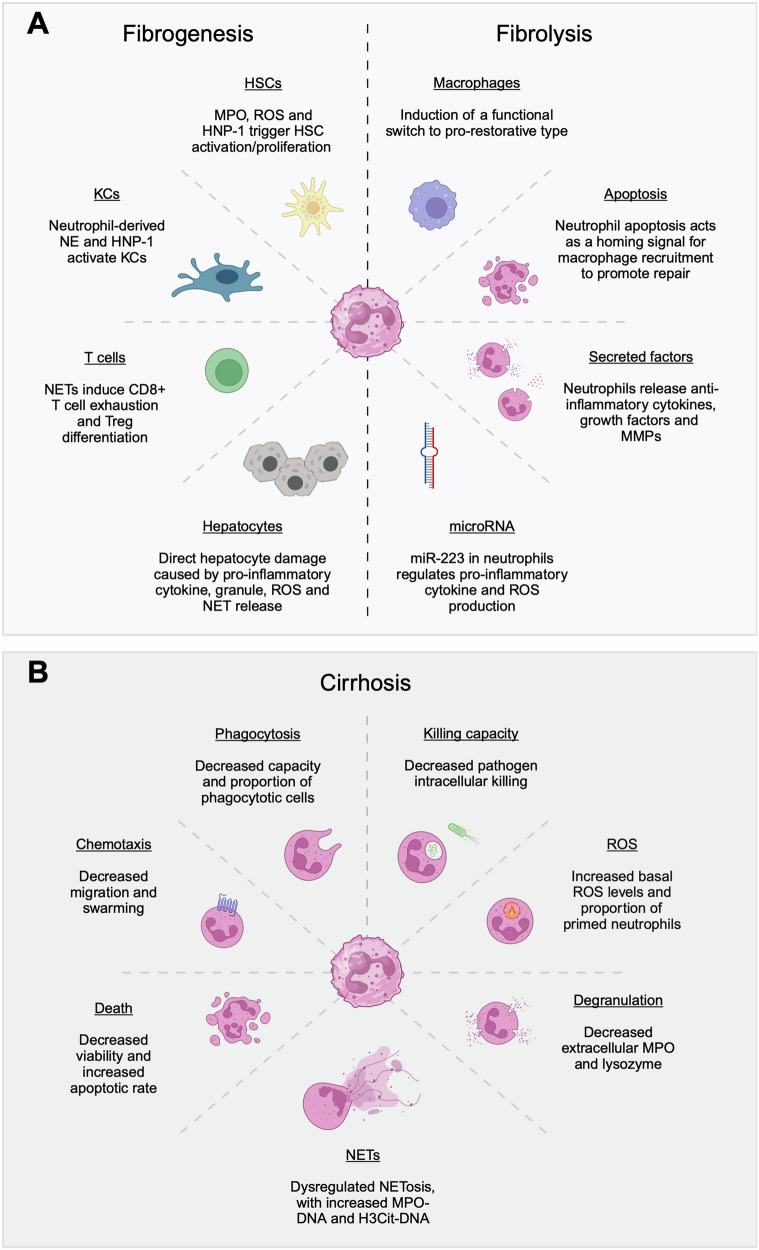
Neutrophils in fibrosis and cirrhosis. **A** A schematic overview of the intricate interactions between neutrophils and other cells in the liver during fibrogenesis and fibrolysis across different chronic liver disease etiologies. **B** A schematic overview of the dysregulated effector functions of peripheral neutrophils in patients with cirrhosis. Abbreviations: HNP-1 human neutrophil peptide-1, HSC hepatic stellate cell, H3Cit citrullinated histone H3, KCs Kupffer cells, miR-223 microRNA-223, MMP matrix metalloproteinase, MPO myeloperoxidase, NE neutrophil elastase, NET neutrophil extracellular trap, ROS reactive oxygen species. This figure was created with BioRender (biorender.com)

Although they are driven primarily by HSC activation and transdifferentiation, neutrophils play an active role in sustaining inflammation and thereby promoting liver fibrogenesis (Fig. 2A). As observed in all etiologies of CLD, neutrophils are recruited in the context of liver inflammation, contributing to fibrogenesis through the activation of KCs and the recruitment of other leukocytes, as shown in mouse models [125–127]. These processes are driven by a combination of their degranulation, ROS production, and NETosis. Neutrophil cytokine production is also implicated in fibrogenesis, with IL-17 released by neutrophils and Th17 cells playing a prominent role in the activation of TGF-β signaling pathways in HSCs [128]. IL-17 sensitizes HSCs to TGF-β by inducing the upregulation of TGF-β receptor II (TGFBR2) while also increasing the activation of downstream kinases [125]. In combination, HSC differentiation into myofibroblasts is induced, ultimately resulting in collagen deposition, thereby aggravating fibrosis.

Emerging evidence suggests that neutrophils also play a critical role in the resolution phase of injury. To successfully promote fibrolysis and degradation of the ECM, the underlying drivers of injury and inflammation must be resolved for HSCs to return to a quiescent phenotype [125]. To encourage this, neutrophils (with/without phagocytic stimuli) have been shown in mice to induce a functional switch in macrophages from a proinflammatory (Ly6C^hi^ CX3CR1^lo^) type to a prorestorative (Ly6C^lo^ CX3CR1^hi^) type [125, 129], which is capable of supporting tissue regeneration. Neutrophil depletion during the restorative phase is associated with ongoing fibrosis [130]. Apoptotic neutrophils can be phagocytosed by macrophages [131], suggesting that neutrophil apoptosis may serve as a cue for injury resolution and tissue repair [103].

Neutrophils also appear to directly promote fibrosis regression (Fig. 2A). In chronic carbon tetrachloride (CCl_4_)-induced injury mouse models, neutrophils have been suggested to contribute to fibrolysis and vascular regrowth [132] through the release of anti-inflammatory cytokines, hepatocyte growth factor (HGF), and anti-fibrotic matrix metalloproteinases (MMPs) 8 and 9 (MMP8 and MMP9). In MASH, extracellular vesicles released by neutrophils induce the downregulation of proinflammatory, profibrotic, and oncogenic hepatocyte genes through microRNA-223 (miR-223), the most abundant microRNA in neutrophils [133, 134]. In keeping with this functional role, miR-223 deletion in AH is associated with increased liver injury in the context of ROS production [135]. miR-223 regulates neutrophil infiltration in steatotic liver disease through the suppression of IL-6 and p47^phox^ expression, indicating that their upregulation contributes to liver injury [130, 136]. Additionally, miR-223 has been suggested to induce the conversion of proinflammatory macrophages to an anti-inflammatory phenotype, thereby restoring liver homeostasis [130, 136].

Cirrhosis: Driving the development of multiorgan failure and death in patients with acutely decompensated cirrhosis (AD) and acute-to-chronic liver failure (ACLF) is increased susceptibility to bacterial infections [137, 138]. This is compounded by the development of high-grade systemic inflammation and immunoparesis, underpinned by defects in both innate and adaptive immune responses [139–142]. A summary of how neutrophil effector functions are impacted in cirrhosis is presented in Fig. 2B. It is postulated that persistent DAMP- and/or PAMP-driven immune cell stimulation in the AD and ACLF states drives systemic inflammation and ineffective responses to intercurrent infections [143, 144]. There is growing evidence that neutrophil antipathogen dysfunction in cirrhosis contributes to poor clinical outcomes, with the NLR shown to be an independent prognostic predictor of survival, irrespective of liver disease severity scores [145].

Neutrophil chemotaxis is reduced in cirrhosis patients because of a combination of intrinsic cellular defects and the impaired or inhibited chemoattractant ability of cirrhotic serum [146]. Neutrophils from cirrhotic patients demonstrate reduced migration toward healthy sera and IL-8 [147], decreased transendothelial migration in response to N-formyl-met-leu-phe (fMLF), increased adhesion to endothelial cells, and altered expression of adhesion receptors [59]. In AD and ACLF patients, CXCL1 and CXCL8 levels are highly elevated, even compared with those in patients with compensated cirrhosis [148, 149]; however, impaired neutrophil migration persists and is associated with adverse outcomes in these cohorts [150]. Overall, the degree of decreased chemotaxis in cirrhosis patients is pathway-dependent and multifactorial.

Impaired phagocytosis capacity has been widely described in patients with cirrhosis. Phagocytic capacity in the context of *E. coli* infection, a common cause of spontaneous bacterial peritonitis, in patients with advanced cirrhosis has been shown to be impaired in whole blood and isolated neutrophils [151]. The number of phagocytic neutrophils has also been shown to be reduced in patients with cirrhosis [152, 153]. In a similar manner, pathogen-killing capacity appears to be diminished in circulating neutrophils, irrespective of their phagocytic capacity [154]. Although impaired intracellular killing of bacteria and fungi (e.g., *Candida albicans*) [155] has been described in the literature, this has not always been replicated, with some studies reporting no change, albeit in the context of different cirrhosis etiologies.

Impaired neutrophil ROS production and degranulation have been described in cirrhosis [151, 156], with varying alterations in capability, production, and function reported [59]. There is an expanded pool of neutrophils with elevated “resting” oxidative burst potential in cirrhosis [151, 157, 158], although a consensus regarding basal ROS production — outside of the hyperinflammatory ACLF state, where it is increased [157, 159]—has not been reached. Cirrhotic neutrophils respond to low physiological stimuli such as fMLF [151, 156, 158], suggesting prepriming from persistent systemic inflammation. However, in patients with active infection, ROS production has been shown to be unaltered in response to fMLF or *E. coli* stimulation. The number of neutrophils that produce ROS in response to more potent stimulation, such as *E. coli*, has been reported to be unchanged [151, 153] or decreased in cirrhosis [158]. Importantly, however, the level of intracellular ROS is decreased, suggesting the possibility of immune exhaustion secondary to persistent inflammation [59].

The intracellular neutrophil enzyme content and its release from neutrophil granules upon stimulation and activation have been shown to be reduced in patients with cirrhosis. The number of cirrhotic neutrophils that produce MPO, as well as the total MPO produced, has been reported to be reduced [160]. However, other studies suggest that MPO’s extracellular release [154] or end function is impaired [161] rather than being the total initially produced. Currently, there are limited observations to support variation in the cytokine production capability of neutrophils in cirrhosis [159], particularly given the generally low levels of cytokines produced by neutrophils.

NET formation has been implicated in sustained liver injury and inflammation. Dysfunctional NETosis and impaired clearance of apoptotic neutrophils (efferocytosis) following binge drinking in mouse models of AH have been shown to exacerbate liver injury associated with sepsis [109]. NET formation in response to infection, fMLF, and PMA is elevated in patients with compensated cirrhosis and ACLF [159]. Elevated NET markers such as H3Cit-DNA and MPO-DNA have been found in cirrhosis and ACLF [162, 163], and plasma from patients with decompensated cirrhosis and ACLF has been shown to induce NET formation in healthy control neutrophils [164]. NETs remain a relatively novel area of investigation in cirrhosis pathogenesis. At present, there is a limited understanding of the role of neutrophil death in cirrhosis. Although some studies have suggested that the viability of neutrophils is reduced [165] and the rate of apoptosis is increased [166], this phenomenon has not been widely explored.

##### HCC

HCC is the most common primary liver cancer and typically develops in the context of underlying CLDs of various etiologies. Although liver carcinogenesis is a multifactorial process, with predisposing factors varying depending on the cancer subtype [15], chronic inflammation is believed to be central to tumor pathogenesis, driving development, progression, and metastasis [167]. As such, recognition of the complex immunological landscape underpinning HCC pathogenesis is critical for the development of targeted immunotherapies [168], particularly as disease progression limits the number of therapeutic options available for HCC treatment [169]. In the context of carcinogenesis, the immune-regulatory role of the liver in maintaining tolerance amid persistent inflammatory stimuli is critical for preventing tissue injury while sustaining systemic immune tolerance. This state is characterized by complex interactions between circulating leukocytes and liver-resident cells, as well as the fine regulation of pro- and anti-inflammatory cytokines. As such, the hepatic tumor microenvironment (TME) is unique, with preneoplastic lesions able to bypass surveillance, maintain evasion from cytotoxic lymphocytes, and ultimately develop into HCC tumors [83].

The HCC microenvironment is particularly immunosuppressive, with immune cell infiltrates being primarily composed of heterogeneous tumor-associated neutrophil (TAN) types. Depending on environmental signals, TANs can be polarized into antitumor (N1) or protumor (N2) phenotypes [83]. However, there is significant overlap in their morphology (typically mature with segmented nuclei), cell-surface expression markers (CD10^hi^ CD11b^hi^ CD16^hi^ CXCR2^+^ CXCR4^-^), and functional characteristics [170, 171]. Although various phenotypic markers have been described as differentially expressed between these populations, functional assays are predominantly used to distinguish them. N1 neutrophils exhibit comparatively increased migratory, oxidative burst, and phagocytosis capacity [170, 171], enhancing their cytotoxic, antitumour functions.

Notably, polymorphonuclear myeloid-derived suppressor cells (PMN-MDSCs) constitute an immature neutrophil population (CD33^+^ HLA-DR^−/lo^) that is functionally immunosuppressive [172]. Under the influence of tumor-released growth factors [e.g., granulocyte colony-stimulating factor (GM-CSF)] and cytokines (e.g., IL-6), aberrant granulopoiesis is stimulated, resulting in the expansion of MDSCs. These cells, in turn, can further facilitate disease progression, as they are recruited into the circulation and TME [172, 173]. Within the hepatic TME, PMN-MDSCs progressively increase their protumorigenic capacity—promoting tissue remodeling through MMP and TGF-β release [174] and supporting angiogenesis via vascular growth factor (VEGF) [175]—and exerting their immune-suppressive effects—through inducible nitric oxide synthase (iNOS), cyclooxygenase 2 (COX2), prostaglandin E2 (PGE2), and IL-10 [83]. Although these cells appear to be distinct from the N2 phenotype, typically of low density, it remains unclear whether their differentiation or expansion reflects a specific state of neutrophil maturation or follows an entirely separate trajectory.

Neutrophil recruitment into the tumor and polarization to a pro-tumorigenic type are crucial for tumor development. HCC cells and stromal cells produce chemokines to drive neutrophil recruitment. Various CXC chemokine receptors are upregulated in HCC tumor cells [83], with CXCL2 and CXCL8 released by tumor-associated monocytes [176] and with CXCL12 released by cancer-associated fibroblasts (CAFs) and stromal cells [177, 178]. Neutrophils begin to accumulate in hypoxic regions in HCC via the activity of hypoxia-inducible factors [179]. In HCC, elevated circulating IL-6, GM-CSF, TGF-β, and PGE2 polarize neutrophils to a protumor phenotype. Moreover, circulating factors in the TME may promote further polarization. GM-CSF, in addition to promoting granulopoiesis, may bias the release of MDSCs [83]. Whether neutrophils first undergo polarization to a protumor phenotype in the circulation and are then recruited to the tumor, or vice versa, remains unclear [83]. Upon accumulation, TANs can promote tumorigenesis by creating an immunosuppressive environment, increasing tumor cell proliferation and survival, promoting extracellular tumor matrix remodeling, and stimulating angiogenesis.

Neutrophils suppress antitumoral immune responses [e.g., via programmed death-ligand 1 (PD-L1) expression], inhibit CD4^+^ and CD8^+^ T-cell proliferation, cytokine production, and cytotoxicity, and promote the expansion of regulatory (Treg) cells [180]. TANs also contribute to the recruitment of macrophages and Treg cells through the expression of CCL2 and CCL17 and via TLR4 signaling. This phenomenon has been shown to promote resistance to sorafenib, a tyrosine kinase inhibitor [181], with neutrophil depletion leading to improved efficacy in preclinical models. ROS production inhibits T-cell activation and promotes IL-10 and TGF-β expression by CD4^+^ T cells while suppressing CD8^+^ T cells [182]. Additionally, through the release of nitric oxide (NO) and arginase 1 (ARG1), neutrophils suppress T-cell proliferation and promote apoptosis [183] while impairing NK cell cytotoxicity [184]. Neutrophils may also be implicated in the induction of stem-like cancer cells, which are capable of rapid proliferation and show enhanced survival capacity [185]. These factors, in turn, seem to drive neutrophil recruitment through CXCL5 [185], leading to further tumor propagation.

**Fig. 3:**
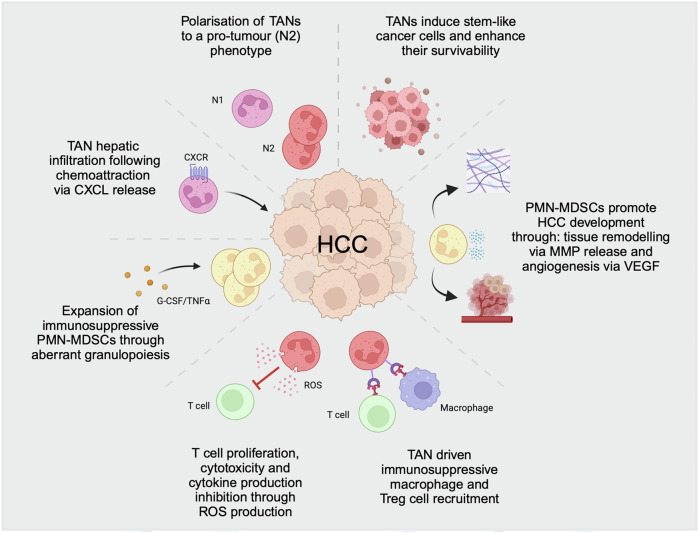
Neutrophils in hepatocellular carcinoma. Schematic overview of the role of neutrophils in the development and progression of hepatocellular carcinoma (HCC). Abbreviations: CXCL C-X-C motif chemokine ligand, MMP matrix metalloproteinase, PMN-MDSCs polymorphonuclear myeloid-derived suppressor cells, ROS reactive oxygen species, TAN tumor-associated neutrophil, Treg T regulatory cell, VEGF vascular endothelial growth factor. This figure was created with BioRender (biorender.com)

The abundance of tumoral and circulating neutrophils has been reported to independently predict adverse outcomes in patients with HCC [186, 187]. The presence of peritumoral neutrophils correlates with metastasis, which is consistent with the expansion of circulating TANs, increasing the dissemination capacity of the tumor [188]. TANs directly enhance the metastatic capability of HCC cells through the release of growth factors (HGF, VEGF), oncostatin M (OSM), and MMP9 [175]. MMP9 stimulates ECM remodeling, HGF/c-MET upregulation, and VEGF angiogenesis, all of which facilitate tumor budding, survival, invasion, and metastasis. Compared with those in nonmetastatic HCC, neutrophils in HCC display increased NETosis capacity, with increased NETs observed in metastatic HCC [189]. HCC cells can internalize NETs, activating TLR signaling and leading to transformation into a more aggressive phenotype, with increased invasiveness and cell survival capability [83, 179]. In summary, neutrophils exhibit diverse functions and are implicated in HCC pathogenesis, from local tumor development and progression to metastasis (summarized in Fig. 3).

### Eosinophils

#### Origin and functions of eosinophils

Eosinophil development, termed eosinophilopoiesis, involves the differentiation of bone marrow hematopoietic stem cells into CMPs, followed by GMPs, before committing to the eosinophil lineage through eosinophil lineage-committed progenitors, which subsequently mature into eosinophils [60, 190]. Notably, heterogeneity in eosinophil lineage commitment has been shown, suggesting that the GMP stage may not be essential for human eosinophilopoiesis, unlike in mice [191, 192]. Eosinophil lineage commitment, differentiation, and maturation are regulated by a synergistic network of transcription factors, including GATA-binding proteins 1 and 2 (GATA-1 and GATA-2), CCAAT enhancer-binding protein alpha (c/EBPa), PU.1 (a member of the ETS family), Friend of GATA-1 (FOG1), and X-box binding protein 1 (XBP1) [190, 193–199]. In addition to transcriptional regulation, cytokines such as IL-3, IL-5, and GM-CSF play critical roles in eosinophilopoiesis [200], with IL-33 being implicated in facilitating eosinophil maturation via an IL-5/IL-5R-dependent mechanism [201].

Eosinophils are recruited via eotaxins such as CCL11 and CCL24, which are recognized by C–C motif chemokine receptor 3. They secrete lipid mediators and several granule proteins, including major basic protein 1 and 2 (MBP-1 and MBP-2), eosinophil cationic protein (ECP), eosinophil-derived neurotoxin, and eosinophil peroxidase [60, 202]. These granules also contain various cytokines and chemokines (e.g., IL-4, IL-6, IL-10, IL-12, IL-13, and TNF-α), contributing not only to the cytotoxic effects of eosinophils on host defense but also to potential tissue damage [60, 202]. Like NETosis, these cells produce eosinophil extracellular traps, which play a role in inflammatory reactions [203]. In addition to their cytotoxic functions, eosinophils are involved in both innate and adaptive immune regulation—for example, they express major histocompatibility complex class II (MHC-II) and costimulatory molecules—as well as in tissue repair. These diverse roles underscore their importance in maintaining homeostasis and contributing to the pathogenesis of various diseases [204, 205].

#### Eosinophils in chronic liver inflammation

##### AIH, PBC, and PSC

Eosinophils are present in the bone marrow, blood, and peripheral organs but are infrequently detected in the liver during homeostasis [202]. To date, few studies have explored the role of eosinophils in noninfectious chronic liver inflammation. Despite some controversy, elevated intrahepatic levels of IL-2, IL-5, IFN-γ, and TGF-β have been reported to be more pronounced in PBC patients than in AIH patients [206, 207]. Increased numbers of eosinophils in the blood and liver of PBC patients, primarily in the vicinity of damaged bile ducts, have been observed and correlate positively with increased IL-5 expression [206–208]. Intriguingly, unlike neutrophils, eosinophilia is more prominent in the early stages of PBC, highlighting the potential of this subset as a diagnostic marker and a target for early therapeutic intervention [208]. Although eosinophil infiltration might contribute via degranulation to early tissue damage, its role in PBC disease progression remains unclear. The first-line therapeutic option for PBC, UDCA, has been shown not only to reduce eosinophil infiltration in the liver but also to inhibit degranulation [209]. Although eosinophilia has been described in AIH blood and PSC livers, minimal evidence exists regarding the role of these cells in their immunopathogenesis [210–212]. One study in children with AIH investigated single-nucleotide polymorphisms in cytokines associated with eosinophil maturation, such as IL-4, IL-5, and IL-13, and suggested a potential link between eosinophils and AIH pathogenesis in pediatric patients [213].

##### Fibrosis

Eosinophils have been implicated in combating parasitic infections such as *Schistosoma mansoni* infection [214, 215], which can lead to liver disease, during which a marked increase in both blood and tissue eosinophil recruitment, accompanied by increased IgE levels, has been observed. This response is mediated by IL-4 and IL-5, which increase eosinophil activation and facilitate parasite elimination [216]. The cytotoxic effects of eosinophils are attributed primarily to two mechanisms: intracellular killing—via phagocytosis and the production of ECP and MBP—and extracellular killing mediated by degranulation [60, 217]. However, these same mechanisms may also contribute to tissue damage. In IL-5 knockout mice infected with *Schistosoma mansoni*, eosinophil depletion and reduced levels of the profibrotic cytokine IL-13 have been observed, suggesting that IL-5 blockade may mitigate liver damage and fibrosis [218]. Hence, these cells play dual roles, both protective and tissue damaging. The significance of eosinophils in liver ischemia‒reperfusion and drug-induced liver injury has recently attracted interest [60]. Eosinophil recruitment to the liver was found in both patients and mice with acetaminophen-induced acute liver injury, where these cells appear to exert a protective effect [219]. Compared with those in WT mice, more severe injury was observed in eosinophil-deficient (DdblGata1) mice, as evidenced by elevated serum ALT levels and increased hepatocyte necrosis. The recruitment of eosinophils to the liver is thought to occur in a macrophage-dependent manner [219–221]. Finally, eosinophil accumulation has also been found to coincide with hepatocyte proliferation during liver regeneration [222].

##### HCC

The role of eosinophils in cancer development is controversial, as they have been demonstrated to play both pro- and antioncogenic roles across different cancer types [223, 224]. Eosinophils generally exhibit antitumoural characteristics in HCC. They directly mediate tumor cytotoxicity when cocultured with MH134 cells (an HCC cell line) in the presence of several mediators, such as IL-5 and CCL11 [225]. In IL-5/CCL11 knockout mice or eosinophil-deficient mice, tumor growth is significantly enhanced [226]. Additionally, the inhibition of dipeptidyl peptidase-4 (DPP4/CD26) promotes eosinophil infiltration into solid tumors by increasing CCL11 levels, leading to significant suppression of tumor growth [227]. Eosinophils may also exert indirect cytotoxic effects through the expression of NK cell surface markers, suggesting that NK cell-like activities are involved in antitumour immunity. Similarly, eosinophils are proposed to interact with various lymphocyte subsets, including NK cells and CD4^+^ and CD8^+^ T cells, under the regulation of cytokines and eotaxins, thereby orchestrating antitumour immune responses [228]. Moreover, clinical studies suggest a potential prognostic role for eosinophils in patients with HCC receiving sorafenib treatment. Low blood eosinophil counts prior to sorafenib administration are negatively associated with overall survival and progression-free survival, further highlighting their relevance in HCC prognosis [229].

### Monocytes and macrophages

#### Origin and functions of monocytes/macrophages

Monocytes are phagocytes derived from hematopoietic stem cells that are typically found in peripheral blood and are able to differentiate into tissue macrophages or DCs during pathological inflammatory conditions [230]. Human peripheral monocytes are classified into three distinct subsets on the basis of the expression of the surface markers CD14 and CD16: classical monocytes (CD14^+^ CD16^lo^), nonclassical monocytes (CD14^lo^ CD16^hi^), and intermediate monocytes (CD14^+^ CD16^+^) [231]. In mice, monocytes are generally described as Ly6C^+^ or Ly6C^−^ cells, corresponding to their classical and nonclassical human counterparts, respectively. Tissue macrophages, such as skin Langerhans cells, lung alveolar macrophages, and hepatic KCs, are considered the first line of defense against pathogens and play significant roles in tissue development and maintenance of tolerance [53]. In mice, many resident tissue macrophage populations arise during embryogenesis, where erythro-myeloid progenitors (EMPs) give rise to premacrophages (p-Macs) and monocytes that seed embryonic tissues [232], subsequently acquiring resident cell phenotypes in response to niche-specific cues and transcriptional activators [233].

**Fig. 4:**
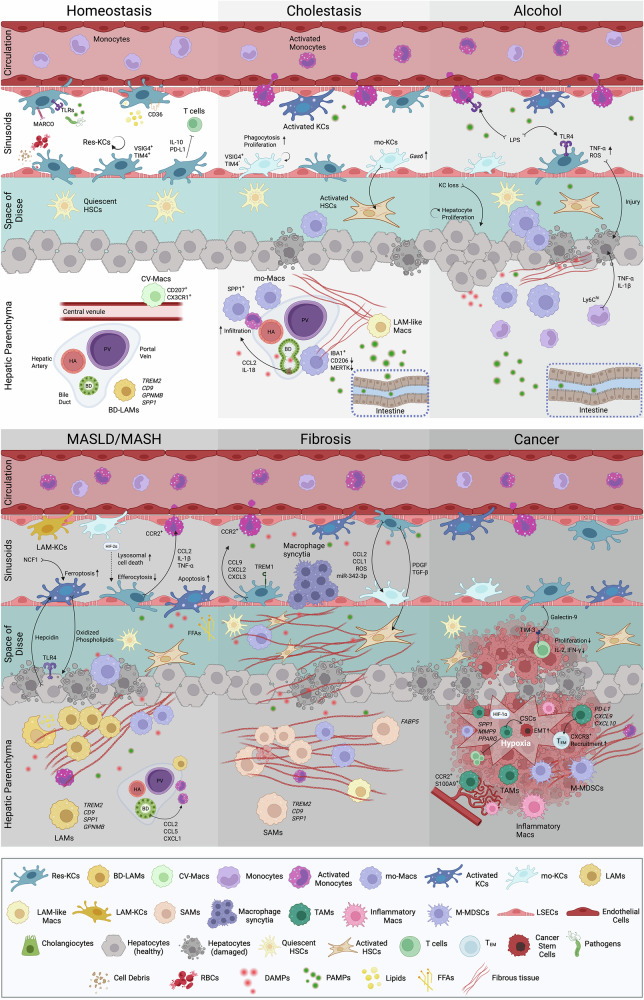
**Macrophages in chronic liver inflammation**. Schematic overview of the roles of hepatic macrophage subsets in homeostasis and different chronic liver diseases. **Homeostasis**: Embryonically derived resident Kupffer cells (Res-KCs) reside within sinusoids, possess self-renewal capacity, and perform various functions, including phagocytosis of pathogens, regulation of lipid and iron metabolism [e.g., removal of red blood cells (RBCs)], clearance of cellular debris, and maintenance of immune tolerance. The following additional liver macrophage subsets derived from bone marrow (BM) monocytes are present: central vein macrophages (CV-Macs) and bile duct lipid-associated macrophages (BD-LAMs). The composition of the hepatic macrophage pool is altered during chronic inflammation. **Cholestasis:** Bile duct damage [e.g., primary sclerosing cholangitis (PSC)] triggers the release of chemoattractants (e.g., CCL2 and IL-18), leading to increased infiltration and periportal accumulation of monocyte-derived macrophages (mo-Macs). In this context, monocyte-derived KCs (Mo-KCs) exhibit potential for communication with HSCs via factors such as Gas6 and increased phagocytosis capacity. The presence of LAM-like macrophages has also been described. **Alcohol:** In alcohol-related liver disease (ALD), increased gut permeability results in increased bacterial translocation and entry of pathogen-associated molecular patterns (PAMPs) into the liver, e.g., lipopolysaccharide (LPS); persistent exposure to these PAMPs activates liver macrophages, causing TNF-α and reactive oxygen species (ROS) production and perpetuating liver injury. Experimental depletion of KCs in mice led to aberrant hepatocyte proliferation, mimicking human disease. **MASLD/MASH:** In metabolic dysfunction-associated steatotic liver disease (MASLD) and metabolic dysfunction-associated steatohepatitis (MASH), a reduction in Res-KC numbers occurs due to impaired self-renewal and overactivation by insults, e.g., free fatty acids (FFAs), that may trigger apoptosis. Furthermore, KCs undergo ferroptosis after hepdicin-induced increased iron uptake, which is stimulated by macrophage-derived NCF1, whereas hypoxia-induced factors such as HIF-2α can promote lysosomal cell death. Mo-Macs are recruited in response to hepatocyte-derived damage-associated molecular patterns (DAMPs) and chemokines released from activated KCs (e.g., CCL2, IL-1β, and TNF-α) and cholangiocytes (CCL2, CCL5, and CXCL1). LAMs of both KC and BM origin emerge and expand, expressing the TREM2, SPP1, and GPNMB markers. **Fibrosis**: Liver inflammation is perpetuated by activated KCs, some of which express TREM1, as well as by CCL2/CCR2-recruited monocytes and mo-Macs. KCs facilitate immune cell recruitment through the production of various chemokines (e.g., CCL9, CXLC2, and CXCL3) and promote HSC activation and differentiation via the production of profibrotic factors (e.g., PDGF and TGF-β). LAM-like Macs are also observed, along with profibrogenic TREM2^+^ CD9^+^ SPP1^+^ scar-associated macrophages (SAMs) and FABP5-expressing macrophages around the fibrotic niche. Syncytial macrophage structures have been shown to play a role in KC in mouse fibrosis models. **Cancer:** Tumor-associated macrophages (TAMs), monocytic myeloid-derived suppressor cells (M-MDSCs), and Res-KCs promote tumorigenesis and dampen immune responses in hepatocellular carcinoma (HCC). CD68^+^ CD11b^+^ CD16^-^ inflammatory macrophages (Inflammatory Macs) and CCR2^+^ S100A9^+^ TAMs accumulate periportally and around irregular blood vessels within tumors. In a hypoxic environment, the expression of matrix metalloproteinases (MMPs) and SPP1 denotes protumorigenic TAMs, which play roles in cancer stemness, epithelial‒mesenchymal transition (EMT) and neovascularization and dampen antitumor responses. PD-L1^+^ TAMs can have antitumour functions by facilitating the recruitment and activation of CXCR3^+^ effector memory T (T_EM_) cells. Abbreviations: CCR chemokine receptor, CCL C-C motif chemokine ligand, CXCL C-X-C motif chemokine ligand, TLR toll-like receptor, LSECs liver sinusoidal endothelial cells. This figure was created with BioRender (biorender.com)

Under homeostatic conditions, the liver macrophage compartment (Fig. 4) is composed almost exclusively of resident Kupffer cells (Res-KCs), which originate primarily from yolk sac-derived EMPs. These progenitors migrate to the fetal liver on embryonic day (E8.5) and give rise to (p-Macs) and fetal liver monocytes. p-Macs, along with some fetal liver monocytes, eventually differentiate into KCs during organogenesis. This differentiation process is significantly regulated by the TGF-β-dependent transcription factor ID3 and the evolutionarily conserved ALK-1/BMP9 axis [29, 53, 61, 232, 234]. KCs are long-lived and have self-renewal capacity, with their identity maintained in part through interactions with LSECs [235], and are linked to the expression of Nr1h3 [gene encoding liver X receptor α], a transcription factor involved in cholesterol processing [236].

KCs are located within the hepatic sinusoids, predominantly in the periportal regions. Their membrane protrusions extend toward the peri-sinusoidal space, enabling close contact with HSCs and hepatocytes. Interplay with these cells, as well as interactions with gut commensals and TLR4/MyD88-mediated LSEC microbial sensing, have been proven crucial for the maintenance of this spatial distribution, which, in turn, is of paramount importance for the preservation of tissue homeostasis [22, 28–30].

The main functions of KCs in homeostasis include the phagocytosis of pathogens, the maintenance of an immune-tolerant microenvironment [28, 37–48], the clearance of erythrocytes, aged platelets, and apoptotic cells [237–239], and the regulation of cholesterol homeostasis via the scavenger receptor CD36 [50, 240, 241]. Immune tolerance is of paramount importance in the liver to prevent tissue damage. Blood entering through the portal vein carries a diverse array of antigens, including food-derived components, commensal microorganisms, pathogens, and microbial byproducts. KCs, along with LSECs and DCs, localized within and around the sinusoids are in a privileged position to recognize these antigens and mount an appropriate, self-limiting immune response [49]. KCs contribute to local immune tolerance through multiple mechanisms: sequestration of PAMPs via scavenger receptors such as MARCO, secretion of anti-inflammatory cytokines [30], passenger T-cell arrest or promotion of local regulatory T-cell proliferation [242], and the expression of regulatory molecules, including immune checkpoints [243].

A multitude of KC markers have been described, reflecting their diverse physiological duties. Both mouse and human KCs express T-cell immunoglobulin and mucin domain containing 4 (TIMD4) and V-set and immunoglobulin domain-containing 4 (VSIG4), which play significant roles in liver-mediated iron homeostasis [244] and phagocytosis [245], respectively. Mouse KCs express the macrophage markers CD64 and F4/80, in addition to C-type lectin domain family 4 member F (CLEC4F) (not detected in human KCs); CLEC4F is expressed relatively late during KC maturation [246–248]. A full consensus has yet to be reached regarding a set of markers that outline a bona fide human KC population. However, the surface markers VSIG4 and folate receptor beta (FOLR2) are increasingly favored, along with other markers, such as CD163 and MARCO [29]. Under certain inflammatory conditions, in mice, bone marrow-derived monocytes are recruited to the liver and may differentiate into monocyte-derived KCs (mo-KCs) [29, 249, 250], but current knowledge does not allow for a clear demarcation between Res-KCs and mo-KCs in humans. Recently, CLEC2 has been proposed as a marker to discriminate between KCs and monocyte-derived macrophages (mo-Macs), as it emerges early during KC development [251].

In addition to KCs, three other macrophage subsets have been described in healthy livers. Liver capsule macrophages (LCMs) are monocyte-derived cells (expressing CX3CR1 and CD207 in mice) lacking KC markers such as VSIG4 [29, 252]. Another subset has been shown to be localized around bile ducts under homeostatic conditions [29] (Fig. 4); these cells express genes such as *Gpnmb*, *Spp1*, *Trem*2, and *Cd9*, resembling the transcriptomic profile of lipid-associated macrophages (LAMs) observed in the fatty liver [253]. Molecular cartography data have also highlighted the presence of a CD207^+^ macrophage subset distinct from LCMs, localized around the central vein, named central vein macrophages [29]. Notably, transcriptomic analyses have revealed the existence of two phenotypically and functionally distinct KC subsets, namely, CD206^lo^ endothelial cell-selective adhesion molecule (ESAM)^−^ KC1s and CD206^hi^ ESAM^+^ KC2s [254, 255]; however, this remains a subject of ongoing debate within the field [29, 256]. As discussed below, the hepatic macrophage landscape becomes more complex in chronic liver inflammation.

#### Monocytes/macrophages in chronic liver inflammation

##### MASLD

In MASLD/MASH, the total number of hepatic macrophages increases due to the recruitment of circulating monocytes, which differentiate into macrophages in the liver (Fig. 4) [50]. KCs exhibit a dysregulated phenotype in MASLD/MASH, resulting in either a direct or a cellular interaction-mediated effect on the disease trajectory [66]. Steatosis-associated factors, such as free fatty acids, can activate KCs via TLR2 and TLR4 [257], and an inflammatory response can also be triggered through DAMP-mediated inflammasome activation. This leads to the release of proinflammatory cytokines such as IL-1β, TNF-α, and CCL2 [258, 259], which act as chemoattractants for peripheral monocytes [260]. CCR2^+^ monocytes are periportally distributed in the livers of patients with MASH, and their abundance is positively correlated with disease severity and the extent of fibrosis [261]. Rapid accumulation of monocytes has also been observed in mouse models of diet- and toxin-induced CLD [262]. Recently, osteopontin (SPP1) has been implicated in the recruitment of inflammatory Ly6C^hi^ monocytes in MCD fibrosis and in two additional mouse models. Coombes et al. showed that SPP1 (which in humans is associated with portal inflammation, fibrosis, and the MASH stage) promotes cholangiocyte chemokine production (e.g., CCL2, CCL5, and CXCL1) via the modulation of the noncanonical NF-kB pathway and affects monocyte recruitment and fibrosis levels, highlighting the complexity of cellular interactions in fibrosis [263].

KC numbers are consistently decreased in mouse MASLD/MASH models, with liver-infiltrating monocytes differentiating into mo-KCs and occupying their place [251, 253, 264]. KC apoptotic death seems to affect total KC numbers, as evidenced by TUNEL staining in mouse MASH livers [264]. MASLD-related stimuli (e.g., free fatty acids, cholesterol, and hepatocyte-derived DAMPs) are contributing factors to increased KC death and influence their role in disease progression [265, 266]. Recently, oxidized phospholipids induced by macrophage-derived neutrophil cytosolic factor 1 were shown to promote hepcidin production by hepatocytes, leading to iron deposition in KCs and subsequent ferroptosis [267]. Moreover, the accumulation of free cholesterol in macrophages following the engulfment of dead hepatocytes triggers lysosomal stress and profibrotic activation in macrophages, particularly in the WD model [268]. Hypoxia has also been implicated in KC (over)activation and death in MASLD, with hypoxia-inducible factor 2 alpha having been shown to mediate increased KC death and impaired efferocytosis through transcription factor EB-mediated lysosomal stress [269]. However, the view that KC activation is a key mediator of MASLD pathology has been challenged by recent in vivo WD studies [253] and observations in obese patients [270], which do not support a traditional KC activation phenotype in MASLD.

In addition, impairment of the proliferative capacity of KCs has been shown to affect KC numbers. A diminished ability to self-renew, accompanied by the emergence of mo-KCs, has been reported in a model of *L. monocytogenes* infection in the context of MALSD [39], as well as in an MCD diet model of steatohepatitis [251], where embryonic (as opposed to monocyte-derived) KCs confer tissue protection through hepatic triglyceride storage. While the loss of KCs has been considered a key driver of monocyte recruitment, recent results from human patient samples and two mouse short-term disease models suggest that monocyte recruitment could be initiated long before the KC niche is vacated—at early stages of the disease—to support the sequestration of lipid-laden hepatocytes [271].

The existence of LAMs and their human analogs is generally considered ubiquitous in MASLD, with their niche-specific localization linked to disease progression. Increased proportions of LAMs in patient livers have been found to correlate with more advanced steatosis [29]. Spatial transcriptomics has revealed that, in the steady state, LAMs are primarily found periportally. However, in human steatotic and mouse WD-induced MASLD liver samples, their distribution shifts, with a higher abundance observed in pericentral regions and steatotic zones [29]. In mice, LAMs express high levels of osteopontin [253], whereas TREM2^hi^ macrophages, termed MASH-associated macrophages, express genes associated with endocytosis, lysosomal degradation, MHC-II antigen presentation, and ECM remodeling [272]. The development of LAMs derived from infiltrating Ly6C^hi^ monocytes has been shown to depend on Egr2, a transcription factor that is increased in mouse monocytes and macrophages in MASH. The long-chain fatty acid-Egr2 pathway promotes the differentiation of hepatic monocytes toward LAMs, whereas Egr2 deficiency drives a KC-like differentiation pathway, which is associated with reduced progression from benign steatosis to fibrosis [273].

There is a growing body of research on the role of TREM2^+^ macrophages in MASLD. In the WD model, mo-KCs, which are abundant during the early stages of disease, are progressively replaced by LAMs during regression and play a significant role in tissue restoration. Both subsets express *Trem2*, which is maintained in LAMs during regression and appears to be crucial in MASH. The absence of TREM2 impairs disease resolution by restricting the emergence of a less inflammatory, collagenolytic LAM population [274]. A similar protective effect has been proposed in the MCD mouse model. In this context, TREM2^+^ macrophages localize around areas of fibrosis, whereas TREM2-deficient macrophages display a decreased lipid-handling capacity, lower viability, and a profibrogenic phenotype [275]. Finally, TREM2 expression on macrophages has been suggested to drive the MASH-resolving effect of bariatric surgery in obese patients by preventing inflammation and augmenting efferocytosis [276]. In addition to TREM2, another receptor relevant to MASLD is macrophage scavenger receptor 1. In MASLD patients, MSR1 is expressed in both KCs and lipid-laden macrophages, and its expression is positively correlated with the degree of steatosis. In a mouse model, Msr1 deficiency mitigated proinflammatory macrophage activation by preventing lipid accumulation, suggesting an overall protective role [277].

##### ALD

One of the hallmarks of alcohol-related liver inflammation is increased gut permeability. As the disease progresses, elevated quantities of gut-derived bacteria, e.g., *E. coli* and bacterial products, pass through the portal circulation and are encountered by KCs [278]. Indeed, KCs from rats in a chronic ethanol feeding model have been reported to be activated, with LPS-stimulated KC-derived TNF-α and ROS being implicated in tissue damage [279]. Chronic exposure to increased concentrations of LPS, due to increased gut permeability, leads to TLR4-dependent NF-kB-mediated activation of macrophages and monocytes in chronic ALD patients [280]. Circulating monocytes also display a dysfunctional phenotype in AH. Monocytes from patients with severe AH presented impaired oxidative burst capacity, which was correlated with diminished NADPH oxidase expression. This defect was associated with an increased risk of infection within two weeks, as well as reduced survival rates at 28 days postadmission [281]. Mirroring the results observed in other CLDs, the infiltration of mo-Macs into the liver aggravates hepatic pathology. This has been reported in a mouse model of chronic alcohol feeding, where infiltration of Ly6C^hi^ monocytes expressing TNF-α, IL-1β, and chemokines such as CCL2 was associated with increased tissue damage [282].

More recently, the role of macrophage CRIg (VSIG4) expression has been highlighted in the context of AH. Compared with WT animals, CRIg-deficient mice developed more severe liver injury after chronic plus binge ethanol feeding, exhibiting significantly greater steatosis and liver inflammation. Additionally, these mice showed a diminished ability to clear gut-translocated *E. faecalis*, creating a setting that could contribute to exacerbated liver disease or systemic infection in patients [283]. Finally, through scRNA analysis, a recent study using a model of concurrent Western diet and alcohol feeding identified the replacement of KCs by monocyte-derived cells, while KC ablation led to loss of liver function and increased hepatocyte proliferation, findings that are consistent with observations in human AH [284].

##### PSC and PBC

Compared with those in control tissues, increased numbers of periportal/peribiliary and fibrotic region-adjacent CD68^+^ macrophages have been observed in human PSC livers (Fig. 4) [285, 286]. The macrophages localized to these areas in PSCs were CCR2^+^, and gene expression analysis revealed that a proinflammatory phenotype predominated in peribiliary mo-Macs. Cholangiocyte insults were identified as the driving factor for the recruitment of peripheral monocytes through the secretion of CCL2 and IL-18, as demonstrated by in vitro experiments in which activated and senescent cholangiocytes were used. Treatment with cenicriviroc (a dual CCR2/CCR5 inhibitor) in this model reduced fibrosis and associated markers such as *Tgfb* and *Acta2* [285]. IBA1 expression has been described as a marker shared by all macrophage subpopulations (CD16^+^, CD163^+^, and CD68^+^) found in PSC tissue samples, indicating that an activated macrophage phenotype is positively correlated with disease progression. In mice, the accumulation of IBA1^+^ CLEC4F^−^ mo-Macs and a decrease in IBA1^+^ CLEC4F^+^ KCs were associated with disease progression in the *Mdr2*^−/−^ model of PSC. Interestingly, IBA1-expressing cells were found near CK19^+^ ductular cells in both *Mdr2*^-/-^ livers and human MASLD/MASH and PSC tissues. Similar histological findings were also observed in liver samples from patients with AH. In mice, IBA1^+^ Macs located near ductular cells presented lower expression levels of IBA1, CLEC4F, CD206, and MERTK [286].

Biliary epithelial dysregulation has been shown to drive the development of scar tissue in a mouse model of congenital hepatic fibrosis, primarily in a macrophage-dependent manner. In this model, homozygous deletion of exon 4 of the *Pkhd1* gene led to the secretion of chemokines, including CXCL1, CXCL10, and CXCL12, by cholangiocytes, resulting in the recruitment of bone marrow-derived macrophages. The recruited macrophages secreted cytokines such as TNF-α, which induced the upregulation of ανβ6 integrin, a local activator of latent TGFβ1, thereby promoting collagen deposition. Notably, integrin ανβ6 levels are correlated with both peribiliary CD45^+^ cell accumulation and portal fibrosis [287].

Analysis of KCs in experimental cholestatic liver injury induced by common bile duct ligation (CBDL) [288] revealed that compared with resident KCs, mo-KCs display increased in vivo and ex vivo proliferation, along with antiapoptotic properties. Additionally, mo-KCs exhibited more pronounced phagocytic and reparative potential. The latter was indicated by increased expression of genes such as *Trem2*, *Cd36*, *Igf1*, and *Lpl*, as well as increased bioparticle phagocytosis ex vivo. In addition, mo-KCs also exhibited greater potential for HSC communication and ECM remodeling through the expression of genes such as *Gas6* and *Mmp12*. Depletion of resident KCs highlighted the protective role of mo-KCs in the CBDL model, as evidenced by reduced necrosis, tissue damage, and fibrosis [288].

In a study using two models of experimental PSCs (0.1% DDC-containing diet or CBDL), monocyte infiltration was prominent, while both CLEC4F^+^ TIM4^+^ Res-KCs and CLEC4F^+^ TIM4^−^ mo-KCs were found to be activated in the early stages, exhibiting increased proliferation after 2 days and upregulation of *Tnfα* and *Ccl2* after 4 days of injury. In both models, liver-resident KC numbers decreased, with apparent KC death demonstrated by positive TUNEL staining of CLEC4F^+^ cells. Depletion of CLEC4F^+^ KCs at the onset of PSC did not affect disease progression or myeloid cell infiltration at the end stage [289]. In a subsequent study, the same team reported that in CBDL mice, mo-Macs upregulated *Spp1*, *Trem2*, and *Gpnmb*, resulting in an LAM-like phenotype. Compared with TIM4^+^ KCs, mo-Macs expressed higher levels of *Spp1, Il1b*, *Tnfa*, and *Tgfb* 4 weeks after CBDL surgery. Immunofluorescence data indicated that Spp1+ mo-Macs accumulated near fibrotic regions in both human PSC samples and experimental models (CBDL or Mdr2^−/−^ animals). The functional relevance of Spp1 was highlighted in the CBDL model, where genetic knockout led to the development of a proinflammatory milieu [290]. Additionally, in a recent bulk RNA-seq study of patient liver tissue, a PSC-specific profibrogenic gene signature was identified, implicating *SPP1* along with pathways such as the Wnt and PI3K/AKT signaling pathways in the development of biliary fibrosis [291]. Finally, a significantly increased abundance of immune-suppressive MARCO^+^ cells was recently described in human PSC liver samples compared with healthy controls, suggesting a possible correlation with disease progression [30].

##### Fibrosis

Macrophage-derived triggers can contribute to HSC differentiation toward a myofibroblast-like phenotype [292]. Profibrotic cytokines such as KC-derived TGF-β and platelet-derived growth factor (PDGF) [293, 294], along with proinflammatory cytokines such as IL-1β and TNF-α, and chemokines such as the CCL2/CCR2 and CCL1/CCR8 axes [295], as well as inflammatory mediators (ROS and iNOS) [296] and microRNAs (e.g., miRNA-342-3p) [297], can directly promote HSC differentiation, proliferation, and ECM production. There is a growing body of work aimed at elucidating monocyte and macrophage heterogeneity in liver fibrosis and cirrhosis. Monocyte infiltration of the liver is prominent during fibrosis, with chemokine signaling (CCL2/CCR2) playing a significant role [50, 261]. In addition to the bone marrow, the spleen can act as a reservoir of immune cells for a rapid response to inflammatory conditions. In CCl_4_-induced fibrosis in mice, splenic Ly6C^hi^ CX3CR1^lo^ monocytes have been shown to migrate specifically to the fibrotic liver in large numbers and localize around the central vein within the hepatic lobules, thereby exacerbating fibrosis and increasing hepatic proinflammatory cytokine levels. Interestingly, this also led to the mobilization of endogenous hepatic CX3CR1^+^ cells, which displayed greater somatic motility and membrane indices of cellular crosstalk. In support of these findings, splenectomy mitigated these effects [298].

Monocyte-derived, CD9^+^ TREM2^+^ SPP1^+^ scar-associated macrophages (SAMs), which can promote collagen production by primary HSCs in vitro, are expanded in both human and mouse fibrotic livers, particularly around fibrotic regions [299]. In the same study, a reduction in a TIMD4^-^ MARCO^+^ KC subset was observed, although no significant difference was noted in the total number of KCs between healthy and cirrhotic livers [299]. These results were corroborated by another study, which reported that tissue monocytes were abundant around the portal triad during the early stages of human liver fibrosis, whereas KCs, SAMs, and tissue monocytes populated the portal area at later stages [300]. Additionally, a monocyte-derived SPP1^+^ GPNMB^+^ FABP5^+^ cell subset within the CD9^+^ TREM2^+^ population has been found to accumulate around fibrotic lesions in both human samples and mouse CCl_4_ or HFD-induced liver fibrosis models. Blockade of TGF-β, IL-17A, or GM-CSF in the CCl_4_ model reduced the accumulation of this subset and was associated with decreased fibrosis. On the basis of these findings and supporting transcriptomic data, the authors propose a profibrotic function for this macrophage population [301].

In addition to infiltrating mo-Macs (Fig. 4), KCs have been shown to actively promote hepatic inflammation and fibrogenesis in experimental CCl_4_ fibrosis. TREM1 expression on CD11b^-^ F4/80^+^ cells was found to be upregulated early and maintained throughout the disease course and was associated with increased tissue damage [302]. Interestingly, TREM1^+^ macrophages have also been identified in liver samples from patients with severe fibrosis. In this context, TREM1^+^ KCs release proinflammatory cytokines, facilitate the recruitment of inflammatory cells via CCL9, CXCL2, and CXCL3, and promote fibrogenesis through TGF-β-mediated KC-HSC crosstalk [302]. Remodeling of the KC niche during fibrosis, such as sinusoidal strictures and the formation of collateral vessels, can lead to a loss of KC identity [40]. In the CCl_4_ model, it was recently proposed that KCs transdifferentiate, losing expression of TIM4 and VSIG4, and consequently display impaired functions, including phagocytosis and bacterial killing. Infiltrating monocytes have been shown to form aggregates that acquire KC-like functions, a process driven by the intestinal microbiota. CD44 upregulation on intrahepatic vessels promoted monocyte adhesion and CD36-dependent formation of syncytial structures capable of capturing bacteria within larger vessels. These macrophage syncytia have also been reported in human liver disease [40], although these findings have not yet been independently replicated, and their functional significance in CLD remains unclear.

Hepatic macrophages have also been implicated in the regression of fibrosis, notably through the production of ECM-degrading MMPs, for example, via KC-derived MMP9 [303], and the secretion of Treg-inducing anti-inflammatory cytokines, such as IL-10 and IL-12, which are key in modulating resolution [304]. During peak fibrosis regression in the CCl_4_ model, flow cytometric analysis revealed that the recruited Ly6C^lo^ subset constituted the dominant liver macrophage subpopulation. These cells exhibited a highly phagocytic and restorative phenotype while also expressing genes involved in ECM degradation (Mmp9 and Mmp12). Interestingly, this subset was shown to be derived from Ly6C^hi^ monocytes. Depletion of Ly6C^lo^ cells resulted in diminished scar remodeling capacity, whereas experimental induction of a phagocytic phenotype in vivo augmented fibrolytic activity [305].

A recent study corroborated the previously established importance [274] of TREM2 in macrophages for fibrosis resolution. Specifically, the recruitment of monocyte-derived LAMs occurs in both HFD- and CCl_4_-induced fibrosis, and a subset of both resident and monocyte-derived KCs adopts an LAM-like phenotype localized near injured tissue. This LAM phenotype appears to be driven by efferocytosis, as indicated by ex vivo and in vitro experiments. TREM2 was found to regulate efferocytic capacity, and TREM2 deficiency impaired tissue repair [250]. In addition, ferroptotic death of recruited “inflammatory” macrophages has been observed in CCl_4_ and CBDL models [306], whereas necroptotic death of mo-KCs in a MASLD model [307] was similarly associated with fibrosis mitigation. These findings underscore the pivotal role of recruited macrophages in directing the disease trajectory toward either progression or regression.

Cirrhosis: As discussed above, a significant proportion of patients with cirrhosis may progress to ACLF, which is characterized by peripheral monocyte dysfunction and an increased risk of bacterial infections [137, 138]. MDSCs contribute to dampening systemic immune responses, and their relevance to liver disease has been reported in several studies [308, 309]. In human ACLF, a significant expansion of CD14^+^ CD15^−^ CD11b^+^ HLA-DR^−^ monocytic MDSCs (M-MDSCs) was observed in the peripheral blood of patients with cirrhosis, which was correlated with disease prognosis. M-MDSCs cocultured with T cells resulted in reduced T-cell proliferation. M-MDSCs exhibited a diminished capacity for the production of proinflammatory cytokines (TNF-α and IL-6) after LPS stimulation as well as reduced bacterial phagocytosis. Interestingly, ex vivo TLR3 stimulation led to the upregulation of HLA-DR and partially restored M-MDSC function [310].

The expression of the receptor protein tyrosine kinases AXL and MERTK, as well as checkpoint receptors on monocytes and macrophages, is indicative of a tolerogenic or prorestorative type [311]. AXL^+^ highly efferocytic mature monocytes are expanded in the peripheral blood of patients with cirrhosis, which is correlated with complications and poor outcomes. These cells exert a T-cell-suppressive function and exhibit diminished TNF-α and IL-6 production [312]. AXL is broadly expressed on liver macrophages during homeostasis; however, in cirrhosis, AXL expression is downregulated in response to HSC-derived growth arrest-specific 6. This reduction was highlighted in both the CCl_4_ model and in human cirrhotic liver samples, paralleling disease progression [313]. Monocytes from patients with cirrhosis also express MERTK in a manner that reflects disease severity. Furthermore, MERTK^hi^ CD163^hi^ macrophages are significantly expanded in the livers of ACLF patients, particularly around hepatic sinusoids [314, 315].

Decreased HLA-DR expression, upregulation of IL-10, and an anti-inflammatory transcriptomic profile are associated with a defective antibacterial response (phagocytosis, oxidative burst) in ACLF monocytes ex vivo [316]. Given the involvement of checkpoint molecules in CLD, the programmed cell death protein 1 (PD-1) axis has been shown to be crucial for the function of monocytes/macrophages in this context. Compared with those from controls, human liver macrophages, as well as peripheral monocytes from patients with cirrhosis, overexpress PD-L1, while monocyte PD-L1 is correlated with disease severity and infection incidence [317]. Mouse and human liver macrophages displayed diminished in vivo phagocytosis of *S. aureus* particles and 99mTc-phytate colloids, respectively. In a DDC mouse model, PD-L1 blockade improved macrophage function, reducing the expression of immunosuppressive markers (MERTK, MARCO, and PDGF-β) and enhancing bacterial phagocytic capacity [317].

Recent transcriptomic analyses have provided new insights into monocyte heterogeneity in cirrhosis. Circulating monocytes from patients with compensated and not acutely decompensated (NAD) cirrhosis were partitioned into seven clusters on the basis of scRNA-seq data. Interestingly, a shift toward a phagocytic phenotype in compensated cirrhosis and the emergence of M-MDSCs in NAD were observed [318]. In another study focusing on ACLF, two distinct functional and metabolic profiles of circulating monocytes, termed recovery (R) and nonrecovery (NR) monocytes, were described on the basis of patient outcome. ACLF-R monocytes highly expressed resistin and S100A8/9/11 proinflammatory alarmins and were characterized by a tolerant state with reduced phagocytic capacity. On the other hand, ACLF-NR monocytes appeared activated, overexpressing regulators of inflammasome activation (VIM and SGK1) and genes involved in antigen presentation and antibacterial responses (HLA-A/B, CD74, B2M, LYZ). Metabolic states were also found to be significantly dissimilar between the two monocyte groups, with ACLF-NR showing lower levels of metabolites such as valine, glycine, α-ketoglutarate, and adenosine, indicating reduced activity in the pentose phosphate and leukotriene synthesis pathways [319].

##### HCC and iCCA

The predominant hypothesis is that tumor-associated macrophages (TAMs) and MDSCs act in a protumorigenic manner in HCC [167, 320]. While the traditional dichotomy of the peri- and intratumoural areas remains relevant, imaging mass cytometry and single-cell analysis have provided a more granular dissection of the TME. Three distinct niches have been described within HCC TMEs—normal, fibrotic, and cancerous regions—each of which is differentially infiltrated by macrophage subtypes. Normal regions display an abundance of resident KCs, along with a periportal accumulation of CD68^+^ CD11b^+^ CD16^−^ “inflammatory macrophages”. These cells, along with M-MDSCs, are expanded in fibrotic areas (characterized by increased a-SMA and collagen I levels), whereas within Ki67^+^ cancerous regions, inflammatory macrophages surround irregular blood vessels. Notably, ex vivo work has shown that KCs dampen CD8^+ T-cell^ activation. Furthermore, in vivo depletion of KCs in an experimental c-Myc/NRAS HCC model increased intratumoral T-cell infiltration and reduced the number of PD-1^+^ CD8^+^ cells [321].

PD-L1 expression is generally associated with HCC immune suppression [322]. Interestingly, in an analysis of HCC patient liver tissue, PD-L1 expression on tumor cells was indeed found to be negatively associated with overall and relapse-free survival, but notably, PD-L1 expression on CD68^+^ macrophages positively correlated with overall survival [323]. In patient samples, PD-L1^+^ ICOS^+^ MDSCs and TAMs were found to infiltrate MASH-HCC tumors, colocalizing both peritoumorally and intratumorally with PD-1^+^ CD8^+^ T cells. CD4^+^ and CD8^+^ T cells from MASH-HCC exhibited diminished cytolytic capacity, a phenotype not observed in viral HCC, suggesting an etiology-dependent mechanism of TAM/MDSC-mediated suppression [324]. Furthermore, CSF1R^+^ PD-L1^+^ TAMs were shown to colocalize with MAIT cells in a human HCC cohort, appearing to induce a dysfunctional phenotype marked by impaired tumor infiltration and cytotoxicity. Coculture experiments revealed a cell contact-mediated effect, and PD-1/PD-L1 axis blockade in vitro and in two tumor-bearing mouse models restored MAIT cell IFN-γ production and enhanced MAIT cell tumor infiltration, respectively [325]. Recently, scRNA-seq analysis revealed a subset of CXCL10^+^ PD-L1^+^ TAMs that was positively associated with the response to immunotherapy. These macrophages expressed genes involved in T-cell recruitment (e.g., CXCL9 and CXCL10) and IFN-γ signaling (e.g., STAT1, indoleamine-2,3-dioxygenase 1 (IDO1), and GBP1), and CellChat analysis predicted their involvement in CXCR3^+^ effector-memory T-cell recruitment and activation in the TME [326]. Another checkpoint implicated in the role of macrophages in HCC is TIM-3. KCs in HBV-related HCC have been shown to express galectin-9, a ligand for TIM-3. Galectin-9^+^ KCs colocalized with TIM-3^+^ T cells, which exhibited diminished proliferative capacity and a senescent phenotype. In vitro blockade of this interaction enhances T-cell proliferation and increases IL-2 and IFN-γ production [327].

In humans, CCR2^+^ S100A9-expressing TAMs have been observed at the HCC border and colocalize with newly formed CD31+ blood vessels (Fig. 4). In contrast, TAMs located at the center of tumors appear to be immunosuppressive, as indicated by CD163 expression. In a fibrosis-HCC model (diethylnitrosamine and 16 weeks of CCl_4_ injections), three tumor-associated myeloid populations were isolated and characterized by cell sorting and mRNA profiling. Gr1^lo^ MHC-II^hi^ CCR2^+^ TAMs (TAM1) are highly inflammatory (*Il-1β*, *S100a9*) and angiogenic (*Vegfa, Mmp9*). Gr1^lo^ MHC-II^lo^ (TAM2) expressed Ccl6, Arg1, Mrc1, and NfkBia, displaying an anti-inflammatory phenotype. This population overlapped slightly with the Gr1^hi^ MHC-II^lo/int^ (MI) subset, which also expressed Ccl1 and Ccr2. Inhibition of CCL2 led to reduced vascularization and hepatic blood volume, a trend toward decreased tumor volume, and the presence of nodules with central necrosis. These changes corresponded with a reduction in the Gr1^lo^ MHCII^hi^ CCR2^+^ subpopulation [328].

Comparative transcriptomic analysis of human HCC, normal liver tissue, and fetal liver revealed the presence of distinct macrophage populations in tumor tissue, initially subdivided by high (TAM1s) or low (TAM2/3 s) expression of CD163. Further analysis revealed FOLR2^+^ TAM1s, SPP1^+^ TAM2s, and metallothionein 1G (MT1G)-enriched TAM3s, which were supported by flow cytometric and RNA-FISH data. Notably, TAM1s and TAM2s exhibited distinct spatial localizations within the tumor tissue. Interestingly, TAM1s presented increased expression of hairy and enhancer of split-1 and displayed similarities with fetal liver macrophages, suggesting the acquisition of a fetal-like phenotype with a possible role of Notch signaling in this transition [329].

On the basis of single-cell transcriptomic data, macrophages expressing TREM2 accumulate within HCC tumors. TREM2^+^ macrophages display M2-like polarization [330] and are associated with an anti-inflammatory function but exhibit the capacity to differentiate into protumorigenic MMP9^+^ macrophages [331]. SPP1 and MMP expression have recently been described to define protumorigenic TAM subsets. SPP1^+^ MMP9^+^ macrophages sorted from primary HCC tumors were found to promote HCC cell migration ex vivo, as well as tube formation by human umbilical vein endothelial cells, indicating the promotion of angiogenesis. PPAR-γ was found to be crucial for both the differentiation and protumorigenic effects of these macrophages [331]. HCC patients with increased numbers of cancer stem cells also displayed an accumulation of a HIF-1α-driven SPP1^+^ macrophage subset characterized by the expression of MMPs (MMP9, MMP12, and MMP17), which may enhance cancer epithelial‒mesenchymal transition. These cells are correlated with poor patient prognosis and an inadequate response to immunotherapy, as evidenced by CD8^+^ T-cell exclusion from areas of SPP1^+^ macrophage clustering, as well as suppression of immune-related pathways such as antigen processing and presentation, as indicated by scRNA analysis [332].

In accordance with most data from HCC, TAM infiltration in iCCA has been correlated with immune evasion, worse patient prognosis, and a diminished antitumor response [333, 334], with PD-L1 expression on CD68^+^ macrophages predicting an unfavorable prognosis [335]. The presence of CD86^+^ CD206^+^ TAMs has been shown to serve as a prognostic marker for iCCA [336], whereas the presence of CD163^+^ macrophages was correlated with worse overall survival in a retrospective study of human tumor samples [337]. In another iCCA cohort, neovascularization, regulatory T-cell infiltration, and poor disease-free survival were correlated with CD163^+^ macrophages [338]. A recent multiomics analysis of iCCA tissue revealed three novel molecular subtypes (chromatin remodeling, metabolism, chronic inflammation), each with a distinct prognosis [339]. TAMs, and more specifically APOE^+^ C1QB^+^ macrophages, were significantly overrepresented in the chronic inflammation subtype, whereas a high APOE^+^ C1QB^+^ TAM signature score was correlated with worse overall survival. In a mouse model in which CSF1R blockade was used to deplete TAMs, reduced TNF-α production by T cells was observed, indicating a dampened T-cell-mediated inflammatory response [339].

### Dendritic cells

#### Origin and functions of dendritic cells

DCs are professional APCs with predominant MHC-II expression that sense pathogens through PRRs and orchestrate immune responses by connecting innate and adaptive immunity [340]. They are classified as conventional DCs (cDCs) or plasmacytoid DCs (pDCs) [340]. The former can be further subdivided into CD103^+^/CD8a^+^ type 1 cDCs (cDC1s) and CD11b^+^ type 2 cDCs (cDC2s). DCs are derived from bone marrow hematopoietic stem cells, which give rise to CMPs, which subsequently differentiate into monocyte‒dendritic cell progenitors and common dendritic cell progenitors (CDPs) [340, 341]. Development from CDPs, the precursors of cDCs (precDCs), an intermediate stage between CDPs and cDCs, migrate from the bone marrow to lymphoid and nonlymphoid organs via the blood and eventually differentiate into cDCs [340, 342]. On the other hand, pDCs complete their development and maturation in the bone marrow, diverging from precDCs and developing directly from CDPs [343]. However, a recent study using clonal tracing of hematopoietic stem cells and Cx3cr1^+^ progenitors suggested that cDC1s are more closely related to pDCs than to cDC2s [343, 344]. While Flt3 ligand (Flt3L) is an essential cytokine that mediates DC development through its receptor Flt3, transcription factors, including interferon regulatory factor 8, basic leucine zipper ATF-like 3 transcription factor (BATF3), and nuclear factor interleukin-3-regulated protein, are also involved in this process [342, 345–348].

Studies using scRNA-seq and high-dimensional protein profiling techniques have revealed markers for DC subpopulation identification and mapped the DC lineage, revealing the complex DC lineage and heterogeneity [346–349]. cDC1s specialize in antigen cross-presentation on MHC-I to activate CD8^+^ T cells in response to viral infections, whereas cDC2s express a wide spectrum of TLRs and are more prone to present antigens to CD4^+^ T cells [341, 350]. Clec9a, which is expressed on cDC1s, contributes to their superior ability to take up dying cells and present antigens to T cells; the deficiency of cross-presentation in mice has been shown to result in failure in priming antiviral or antitumour immunity [350, 351]. Compared with cDC1s, cDC2s are characterized by their expression of various cytokines and their ability to stimulate Th1 and Th17 responses in CD4^+^ T cells [352]. In contrast, pDCs express lower levels of MHC-II and a narrower array of PRRs [345]. Nonetheless, these cells are characterized by their ability to recognize pathogen-derived nucleic acids through high expression of TLR7 and TLR9 and to rapidly produce a large amount of type I IFN in response to viruses [353]. Interestingly, cDC1s express high levels of type III IFN when encountering poly I:C; thus, they are likely to interact with pDCs to promote type I IFN production [350, 354]. Upon stimulation, cDCs migrate to the T-cell zone or lymph nodes, which is primarily mediated by the CCR7‒CCL21 axis, for antigen presentation and T-cell activation [349, 355].

#### Dendritic cells in chronic liver inflammation

Hepatic DCs, along with resident KCs, contribute to immune tolerance within the tissue, partly because of their low phagocytic capacity and limited ability to stimulate T-cell responses, as highlighted by increased IL-10 production [356, 357]. All three DC subpopulations have been identified in both healthy or chronically inflamed human and mouse livers [29, 250, 253, 358, 359]. Recently, spatial transcriptomics analysis has placed cDC1s (*Xcr1*, *Clec9a*) and cDC2s (*Cd209a, Mgl2, Clec10a*) predominantly in the periportal regions [29] of the homeostatic liver, with the former located mainly around the lymphatics and the latter surrounding the biliary tree [360].

##### MASLD

The extent to which DCs contribute to MASLD has yet to be delineated, with data largely indicating contradictory conclusions [67, 72, 361]. Early research suggested that the presence of lipids and their engulfment by DCs in human and mouse livers can affect their immunogenic potential, shifting them toward a proinflammatory state [362]. Both cDC1s and cDC2s, along with pDCs, have been shown to be numerically expanded in various dietary models [WD, choline deficient high fat diet (CDHFD), methionine choline deficient diet (MCDD)] [253, 363–365]. In the MASH model, the expansion of total cDCs coincided with a shift in the ratio between the two subsets due to a relative increase in cDC2s [366] and decrease in cDC1s. Furthermore, in WD-fed mice, hepatic cDC2s exhibited heterogeneous expression patterns of Tbet, Mgl1, and CCR2 [253], whereas the total cDC population was shown to express lower levels of the antimicrobial peptide *Slpi* [359]. In both the two-week MCD diet and 6-month CDHFD models, flow cytometry and scRNA-seq data revealed an expansion of XCR1^+^ cDC1s, which was attributed to increased bone marrow and blood progenitor cell proliferation and was correlated with liver pathology. [364]. DC maturation, activation, and a shift toward a more immunogenic phenotype have been observed in MASH models, with the upregulation of markers such as CD80 and CD86, as well as increased TNF-α, IL-6, MCP-1, and IL-10 production [362, 363, 367]. Chu et al. reported that CCL20, an immature DC chemoattractant, is upregulated in the fibrotic MASLD liver. LX-2 cells (a human HSC line) exposed to fatty acids upregulated CCL20 expression in vitro, suggesting that HSCs might be responsible for the influx of immature DCs in the MASLD liver [368].

Recently, a mechanism through which DCs can influence the progression of HFD-induced steatosis in mice has been proposed; the activation of liver DCs is accompanied by the phosphorylation of liver kinase B1 (LKB1), a kinase that plays a regulatory role in DCs. DC-specific deletion of LKB1 led to increased insulin resistance; increased proportions of hepatic regulatory T cells (Tregs) and Th17 cells; increased expression of Cd36 and fibrotic markers; worsened steatosis; and elevated triglyceride and cholesterol levels. This effect was mitigated by the blockade of IL-17A, while ex vivo, cDC2s from LKB1 knockout mice exhibited increased production of the Th17-promoting cytokines *Il6* and *Il1β*, collectively suggesting that DCs impact MASH pathology through the regulation of hepatic Th17 responses [367]. Finally, a Ly6C^hi^ monocyte-derived F4/80^+^ CD11c^hi^ MHCII^+^ DC subset expressing high levels of CX3CR1 emerged during MCD-induced MASH and produced TNF-α, which correlated with hepatic injury in advanced stages of the model [369].

DCs have also been implicated in MASH regression. In MCD-fed mice, CD11c diphtheria toxin receptor (DTR)-mediated depletion of DCs led to exacerbation of inflammation and fibrosis, as well as delayed resolution of pathology [363]. Notably, the CD11c-DTR system is unlikely to be specific to DCs or particular DC subsets. In addition to recent data describing cDC1s as drivers of pathology, a protective role of CD103^+^ cDC1s against MASH pathology has also been proposed. In *Batf3*^−/−^ mice, which lack cDC1s, feeding a high sucrose diet (HSD) for two weeks caused increased recruitment of inflammatory monocytes, the development of a proinflammatory milieu (characterized by elevated IL-1ra, CCL2, CCL-5, and TNF-α), progression toward steatohepatitis, and alterations in lipid metabolism. However, no effect on collagen deposition was observed in either MCDD-fed or HSD-fed *Batf3*^−/−^ mice [365]. Human data regarding DCs in MASLD/MASH remain limited. Deep immunophenotyping of blood samples from patients with MASH before and after lifestyle intervention revealed a positive correlation of cDC2s (HLA-DR^+^ CD123^-^ CD11c^+^ CD141^−^) with MASH activity, particularly hepatocyte ballooning and lobular inflammation. In contrast, cDC1s (HLA-DR^+^ CD123^−^ CD11c^+^ CD141^+^) and pDCs (HLA-DR^+^ CD123^+^) were inversely correlated with MASH and glucose levels [366].

##### Fibrosis

Research on the role of DCs in humans and experimental fibrosis outside the context of MASLD remains limited [71, 370]. Both cDC subsets, differentiated from peripheral blood monocytes, have been reported in proximity to the fibrotic niche in human cirrhosis [299]. The expansion and activation of DCs have been observed in a mouse thioacetamide model, where depletion of CD11c^+^ cells led to decreased hepatic TNF-α levels [371, 372]. Additionally, ex vivo coculture of CD11c^+^ cells with LSECs enhanced T-cell activation and proliferation [371, 372]. However, these studies relied solely on CD11c as a marker to identify DCs, raising the possibility of contamination by non-DC CD11c⁺ cells. A study by Pradere et al. revealed that cDCs and pDCs play a nonsignificant role in promoting fibrosis [373]. In contrast, an antifibrotic effect has been reported, wherein therapeutic Flt3L-mediated expansion of DCs accelerated fibrosis regression in a CCl_4_ model, partly through upregulation of MMP9 [374]. In support of this, recent in vitro data have shown that DC-derived IL-10 can induce apoptosis, reduce proliferation, and downregulate α-SMA RNA, TGF-β1, and SMAD3 in LX-2 cells, further suggesting a potential antifibrotic role of immature DCs in HSCs [375]. In patients with cirrhosis of various etiologies, analysis of peripheral blood revealed a reduction in DC numbers and an increased pDC/cDC ratio compared with those in healthy controls [376].

##### HCC and iCCA

Mounting a sufficient immune response against tumors is highly dependent on DC-mediated recruitment and activation of tumoricidal lymphocytes [71, 377, 378]. Hepatic DCs are intrinsically programmed to maintain tolerance and have been shown, in vitro, to induce IL-10-dependent hyporesponsiveness in T cells, as well as to promote the generation of CD4^+^ CD25^+^ FoxP3^+^ Tregs [379]. This immunosuppressive effect of hepatic DCs can be further enhanced by the TME, with both tumor-associated cells and proteins influencing DC function. Hepatic CAFs are particularly capable of modulating the TME. One study demonstrated that IDO-producing regulatory DCs could be induced by CAFs via IL-6 secretion in vitro [380]. Additionally, HCC tumor-derived alpha-fetoprotein (AFP) has been shown to disrupt DC metabolism by inhibiting mTORC1 signaling, resulting in defective oxidative phosphorylation, impaired mitochondrial integrity, and a diminished capacity to stimulate T cells in vitro [381]. The expression of immune checkpoint molecules by DCs has also been described in human HCC samples, where tumor-infiltrating DCs express PD-L1 and galectin-9 (ligands for PD-1 and TIM3, respectively), among others [382]. Furthermore, PD-1 was found to be overexpressed on peripheral blood mDCs and pDCs from HCC patients [383]. In the periphery, a CD14^+^ CTLA4^+^ DC subset present in the peripheral blood of HCC patients was able to suppress T-cell responses via IDO in addition to IL-10, inducing systemic immunosuppression [384].

Studies on the effects of pDCs in liver cancer are limited [385], but existing data show a correlation with poor prognosis. The infiltration of tumor tissue by pDCs has been associated with worse overall survival, a shorter time to relapse, and increased tumor invasion in both HCC [386, 387] and iCCA [388], as well as with ICOS-ICOSL-mediated activation of a CD4^+^ FoxP3^−^ IL-13^−^ IL-10^+^ Tr1 regulatory cell subset that can suppress the antitumor response [389]. Vaccines targeting DC function in HCC are currently in various stages of clinical and preclinical development; however, this topic is beyond the scope of this article and has been recently reviewed elsewhere [390].

## Future perspectives

On the basis of the latest insights in the field, a substantial amount of information is still lacking regarding the functional and phenotypic heterogeneity of myeloid cells in the liver, particularly in humans. Two key examples that highlight efforts to bridge this knowledge gap involve recent observations of macrophages and neutrophils. Under both steady-state and inflammatory conditions, hepatic macrophages are highly heterogeneous, comprising various niche- and context-instructed subsets (resident and recruited) with distinct transcriptional profiles and activation states. Future studies employing targeted approaches to modulate specific subsets and/or genetically manipulate expressed factors are needed to elucidate their precise functions and contributions to CLD pathophysiology and identify targets for the development of macrophage-tailored therapies. Regarding neutrophils—cells once considered largely transcriptionally inactive—advances in sequencing technologies adapted to their volatile nature have revealed a new level of complexity, including a dynamic transcriptional program that remains to be fully explored in different CLD settings.

The acquisition of this wealth of new information can be attributed, in part, to advancements in novel multiomics approaches. The integration of high-resolution microscopy with proteomic and single-cell or single-nucleus transcriptomic analyses continues to shed light on the functional and phenotypic variations of myeloid subpopulations and to facilitate the discovery of unrecognized subsets through the identification of novel markers. Hence, researchers are now equipped to study cell subsets that have previously remained elusive and to implement a more granular approach in analyzing the involvement of specific pathways or molecules in various forms of CLD. These new approaches may also help overcome challenges associated with studying rare cell populations by enabling more precise molecular and functional distinctions from phenotypically similar cells.

The discovery of novel cell subset markers will be particularly crucial for advancing DC research. The broad use of CD11c-targeted modifications in vivo (e.g., CD11c-cre, CD11c-DTR) does not allow for a more detailed study of the intricacies of pDC or cDC subset biology and their disease relevance. Moreover, DC subset-specific transgenic models (e.g., XCR1-cre) have yet to be employed in the context of CLD. Although DC-targeted therapeutic approaches are already being explored, particularly in oncology with DC vaccines and TME modulation, targeting T-cell/DC or LSEC/DC interactions may also represent promising avenues for antifibrotic therapies.

Finally, spatially resolved omics will further contextualize the role of cell type localization in CLD progression and regression. The spatial distribution of immune cells during homeostasis is vital for supporting their function and often reflects it. As discussed above, the niche-specific accumulation of immune subsets has been linked to various types and stages of CLD, with transcriptomic data providing an additional layer of insight into their function and potential for therapeutic targeting. In the coming years, the broader application of spatial omics to human liver tissue holds great promise for advancing our understanding of liver myeloid cell biology and identifying novel therapeutic strategies.
